# High CD90 (THY-1) expression positively correlates with cell transformation and worse prognosis in basal-like breast cancer tumors

**DOI:** 10.1371/journal.pone.0199254

**Published:** 2018-06-27

**Authors:** Aline Ramos Maia Lobba, Ana Claudia Oliveira Carreira, Otto Luiz Dutra Cerqueira, André Fujita, Carlos DeOcesano-Pereira, Cynthia Aparecida Bueno Osorio, Fernando Augusto Soares, Pranela Rameshwar, Mari Cleide Sogayar

**Affiliations:** 1 NUCEL (Cell and Molecular Therapy Center), Internal Medicine Department, School of Medicine, University of São Paulo, São Paulo, Brazil; 2 Biochemistry Department, Chemistry Institute, University of São Paulo, São Paulo, Brazil; 3 Department of Computer Science, Institute of Mathematics and Statistics, University of São Paulo, São Paulo, Brazil; 4 Department of Anatomic Pathology, A. C. Camargo Hospital, São Paulo, Brazil; 5 Department of Medicine, Rutgers New Jersey Medical School, Newark, New Jersey, United States of America; University of South Alabama Mitchell Cancer Institute, UNITED STATES

## Abstract

Breast cancer is the most prevalent cancer among women, with the basal-like triple negative (TNBC) being the most agressive one, displaying the poorest prognosis within the ductal carcinoma subtype. Due to the lack of adequate molecular targets, the diagnosis and treatment of patients with the TNBC phenotype has been a great challenge. In a previous work, we identified CD90/Thy-1 as being highly expressed in the aggressive high malignancy grade Hs578T basal-like breast tumor cell line, pointing to this molecule as a promising breast tumor marker, which should be further investigated. Here, CD90 expression was analyzed in human breast cancer samples and its functional role was investigated to better assess the oncogenic nature of CD90 in mammary cells. Quantification of CD90 expression in human breast cancer samples, by tissue microarray, showed that high CD90 positivity correlates with metastasis and poor patient survival in the basal-like subtype. The functional genetic approach, by overexpression in the *CD90* cDNA in a basal-like normal mammary cell line (MCF10A) and knockdown in a highly malignant cell line (Hs578T), allowed us to demonstrate that CD90 is involved with several cellular processes that lead to malignant transformation, such as: morphological change, increased cell proliferation, invasiveness, metastasis and activation of the EGFR pathway. Therefore, our results reveal that CD90 is involved with malignant transformation in breast cancer cell lines and is correlated with metastasis and poor patient survival in the basal-like subtype, being considered as a promising new breast cancer target.

## Introduction

Breast cancer is the most commonly detected tumor in females and one of the leading causes of cancer-related death among women in the World [1]. The mammary carcinoma is characterized as a heterogeneous neoplasm, composed of multiple subtypes, which display distinct morphologies and clinical implications, with the ductal carcinoma, originated from the mammary gland epithelium, being the most prevalent one [2, 3]. Clinically, the ductal carcinoma is evaluated according to the expression profile of the estrogen receptor (ER), progesterone receptor (PR) and epidermal growth factor type 2 receptor (HER2) and sub-classified into: hormone-positive receptors (luminal A, Luminal B), HER2-positive and triple negative for hormonal receptors (basal-like) [4]. Generally, tumors expressing the hormone receptors (ER and PR) display the most favorable prognosis, relative to those which only display HER2 or those which do not express any of the three markers (triple negative) [5]. The triple negative basal-like subtype represents approximately 10–15% of all mammary carcinomas, being characterized by high histological grade, high mitotic index and low differentiation [6]. The triple negative subtype has a more aggressive clinical course, when compared with the other subtypes and is associated with a higher risk of distant metastasis recurrence and mortality [7]. Although several studies have been developed in the last few years, due to the lack of proper molecular targets, the diagnosis and treatment of patients with tumors of the basal-like phenotype is still limited and has been a major challenge [5, 8].

Seeking for new breast cancer markers, we previously studied stem cell markers in a panel of breast cancer cell lines representing the different tumor subtypes [9]. We demonstrated that CD90 (a mesenchymal stem cell marker) was highly expressed in the most aggressive basal-like breast tumor cell line (Hs578T), when compared with the non-tumorigenic one (MCF10A) or with the less aggressive lines (MDA-MB-231 and MDA-MB-435) [10], indicating that CD90 expression increased according to the malignancy degree of the cell lines, positively correlating to high malignancy grade in mammary carcinoma [9]. CD90 has also been described in human breast tumor samples in a small subpopulation of CD44^+^/CD90^+^ cells with the basal-like phenotype [11]. This CD44^+^/CD90^+^ cell subpopulation was located on the periphery of tumor nests and adjacent to CD90^+^ stroma, being defined as an invasive tumor front [11]. Furthermore, in the tumorigenic MDA-MB-231 basal-like breast cancer cell line, CD90 was used to identify a highly proliferative and migratory subpopulation of CD105^+^/CD90^+^ cells with “mesenchymal stem cell-like” characteristics, which were described as offering the possibility of a new theoretical basis for breast cancer recurrence and metastasis [12]. Altogether, these results indicated that CD90 is an interesting and promising marker, which should be further studied in order to assess its actual role in human breast cancer.

In the present study, we investigated the relationship between CD90 expression and breast cancer malignacy by two different and complementary approaches: a) assessment of CD90 protein expression in human patient samples using a breast cancer Tissue Microarray (TMA) Array upon ten years of patient follow-up; b) investigation of the functional role of CD90 in basal-like breast cell lines by both gain of function studies, using CD90 ectopic expression in the non-tumorigenic MCF10A cell line and loss of function, in the highly malignant Hs578T triple-negative breast cancer cell line.

## Materials and methods

### Human tissue specimens

#### Tissue Microarray (TMA) casuistics

Primary breast tumor samples were obtained at the A. C. Camargo Hospital, Department of Pathology, São Paulo, Brazil, from patients with invasive ductal and lobular carcinoma. The tissues were collected upon radical mastectomy, modified radical mastectomy or breast-conserving surgery including axillary lymph node dissection. All patients were diagnosed, treated between 1976 at 2005, with clinical follow-up until 2008 at the same Institution, which provided all the clinical information for this work.

A total of 278 patients were selected based on the following criteria: i) tissue sections with the presence of a representative area of invasive lesions, excluding cases with only *in situ* lesions; ii) patients who did not receive neo-adjuvant chemotherapy; iii) in case of patients with relapse, we only included those who had distant metastases. The Institutional Ethics Committee (CIPE- Centro Internacional de Pesquisa e Ensino–A. C. Camargo Cancer Center) approved this study (Number 1813/13) and all patients provided Informed Consent.

#### Immunohistochemistry for TMA and analysis tools

Immunohistochemical staining was performed on tissue microarrays. TMAs were assembled from archival invasive breast carcinoma primary tumor blocks of patients from the A. C. Camargo Hospital (São Paulo, Brazil). For TMA construction, 5mm diameter cylindrical sections of paraffin blocks from biopsy samples from selected patients were carefully cut and mounted in paraffin. Immunohistochemistry for detection of CD90 protein was performed as previously described [13–15]. Briefly, TMA slices (5μm) were adhered onto glass slides and immersed in xylol to remove the paraffin. After rehydration, the tissue peroxidases were blocked with a solution of hydrogen peroxide. The slides were then treated with proteinase K and blocked prior to the addition of primary anti-CD90 antibody (Abcam ab133350) at 1:200 dilution in blocking solution. After incubation for 2h at room temperature, the slides were washed with PBS and incubated for 30min with the proper secondary antibody conjugated to peroxidase. Further washes with PBS were performed to remove the unbound antibodies and signal detection was achieved upon incubation with DAB (diaminobenzidine) for 5min. The slides were then counter-stained with hematoxylin and pre-examined under a light microscope (Carl Zeiss). The CD90 protein abundance in the TMAs core was quantified by their brownish color conferred by DAB. Finally, these TMA slides were scanned with a ScanScope AT Turbo image capture system (Aperio ePathology Solutions Inc) and DAB staining intensities (“Positivity” values) were calculated using the “Pixel Count V9” algorithm (Aperio ePathology Solutions Inc).

#### Amplification of the CD90 coding region

For amplification of the human *CD90* cDNA, *Xba*I and *Mlu*I restriction sites were added to the forward (*TCTAG*AATGAACCTGGCCATCAGCATCG) and reverse (*ACGCGT*TCACAGGGACATGAAATCCGT) primers, respectively, for insertion of the *CD90* cDNA into *Xba*I and *Mlu*I sites of the lentiviral vector.

The Hs578T breast cancer cell line cDNA was used as the template for amplification of the coding region for the *CD90* gene. For the amplification reaction, we used 1X *High Fidelity Buffer* (Finnzymes), 2mM dNTP (Fermentas), 1U *Phusion® Hot Start High Fidelity DNA Polymerase* (Finnzymes), 0.5μM forward and reverse primers, under the following conditions: 98°C for 2min; 40 rounds 98°C for 10seg, 72°C for 120min; and 72°C for 5min for extension. The PCR product was analysed in an agarose gel and the fragment corresponding to CD90 (data not shown) was extracted under UV light, purified using Qiaquick Gel Extraction Kit (Qiagen) and cloned into the pLV vector [16].

#### Cell lines establishment

The non-tumorigenic MCF10A breast cell line, generously provided by Professor Dr. Nathalie Cella, was cultured using DMEM/F12 (Gibco, Rockville, MD, USA) supplemented with 5% horse serum (Invitrogen), 20ng/mL epidermal growth factor (EGF) (Gibco), 10μM cholera enterotoxin (Sigma-Aldrich), 10mg/mL insulin (Invitrogen), 5mg/mL Hydrocortisone (Sigma-Aldrich) and 1% streptomycin/ampicillin [17]. The MCF10A-empty vector (pLV) and MCF10A/CD90^+^ cells were maintained in the same medium supplemented (or not) with EGF for the functional assays.

The Hs578T breast cancer cell line, purchased from ATCC, and their derived Hs578T non-target and Hs578T/shCD90 cell lines were cultured in DMEM, supplemented with 10% fetal calf serum (FCS) (Atená Biotecnologia, Campinas, SP, BR) and 1% streptomycin/ampicillin.

Cells were maintained at 37°C in a humidified 5% CO_2_-air atmosphere and were periodically determined to be mycoplasma-free by a multiplex PCR method.

The MCF10A cell line was transduced with the lentiviral empty vector construct (p156RRLsinPPCCMVIns3IRESPRC-eGFP/pLV) or pLV/CD90 using a third generation lentiviral system (Verma, Salk Institute for Biological Studies—CA) according to Tiscornia and collaborators [16]. After the MCF10A cell line transduction, it was detected 5% and 18% of eGFP positive cells for empty vector and pLV-eGFP-CD90 constructor, respectively. The eGFP^+^ subpopulations of the two cell lines were selected by cell sorting.

To inhibit CD90 expression in the Hs578T cell line, we used five different constructs of the Mission shRNA System (Sigma-Aldrich). shRNAs were transduced into the Hs578T cells using the third generation lentiviral system [16]. Cells were selected using 2μg/mL puromycin (Sigma-Aldrich). By flow citometry and qRT-PCR we demonstrated that the TRCN 0000057025 sequence was the most efficient constructor to CD90 knockdown.

#### Total RNA isolation and cDNA synthesis

Total RNA was isolated using the RNeasy kit (Qiagen) following the manufacturer’s protocol, treated with DNaseI (Thermo Scientific) and the quality was assessed for purity by the 260/280nm and 230/260nm absorbance ratios (Nanodrop, Thermo). A total of 1 μg total RNA was used with SuperScript III Reverse transcriptase (Invitrogen) to generate cDNA, as described by Lobba et al. [9].

#### Real-Time Reverse Transcription PCR (qRT-PCR)

Real-Time Reverse Transcription PCR (qRT-PCR) reactions were performed in 96-well plates with an ABI PRISM^®^ 7300 Sequence Detection System (Applied Biosystems) using Power SYBR^®^ Green to detect dsDNA synthesis. The relative gene expression was normalized with respect to reference genes expression, as previously described [9]. Primers for reference genes used in this study are listed in S1 Table [9]. To amplify CD90 were used the *Forward* primers AAGGACGAGGGCACCTACAC and *Reverse* primer GAGGTGTTCTGAGCCAGCAG.

#### Western blot analysis

Subconfluent monolayers cultures growing in 100 mm plates were washed with ice-cold PBS and then lysed with lysis buffer (M-PER® Mammalian Protein Extraction Reagent Therm, Thermo Scientific) and protease inhibitors (Halt Protease and Phosphatase Inhibitor Cocktail, Thermo Scientific). Cell extracts were centrifuged at 14,000g for 15min and the supernatant fraction was then collected and stored at -80°C. The total protein content for each sample was quantified using the Bio-Rad Protein Assay (BioRad, Hercules, CA, USA). Equal amounts (10μg) of proteins from each extract were boiled in Laemmli’s sample buffer containing 5% β-mercaptoethanol for denaturation. Proteins were resolved in a 10% SDS-PAGE, then transferred to nitrocellulose membranes and analyzed by Western blotting. The membranes were blocked for 1h in PBS-0.1% Tween 20 and 5% milk, followed by incubation with primary antibody overnight at 4°C (1:1.000 dilution). The primary antibodies used were: anti-vimentin (V6630, Sigma-Aldrich), anti-E-cadherin (ab53033, Abcam), anti-N-cadherin (ab12221, Abcam), anti-Erk1/2 (4695, Cell-Signaling), anti-p-ERK1/2 (4370, Cell-Signaling), anti-Jnk (9258, Cell-Signaling), anti-p-Jnk (9255, Cell-Signaling), anti-c-Jun (SC1694, Santa Cruz), anti-p-c-Jun (3270, Cell-Signaling), anti-c-Fos (2250, Cell-Signaling), anti-p-c-Fos (5348, Cell-Signaling), anti-EGF-R (3777, Cell-Signaling), anti-p-EGF-R (4267, Cell-Signaling) and anti-β-actin (A5441, Sigma-Aldrich). After three washes of 10min each with PBS-0.1% Tween 20, the membranes were incubated for 2h at room temperature. Immunoreactive proteins were detected with an appropriate secondary horseradish peroxidase-coupled antibody (Cell-Signaling) at 1:2000 dilution. Signals were detected using Supersignal West Femto Maximum Sensitivity Substrate (Thermo Scientific). The membranes were then stripped using Restore Stripping Buffer (Thermo Scientific) and incubated with β-actin antibody (Sigma-Aldrich) as an endogenous protein. Quantitative densitometry was carried out with the ImageJ 1.46r software.

#### *In vitro* cell growth analysis

5x10^3^ cells were seeded onto a 96-wells plate and after 72h the cells were collected. Cell proliferation was determined using CyQuant Cell Proliferation Assay (Invitrogen), as recommended by the manufacturer.

#### Immunofluorescence

Immunofluorescence was adapted from Maria-Engler et al. [18]. Briefly, cells were seeded onto 13 mm diameter glass coverslips and maintained under usual culture conditions until sub-confluence (less than 80%). Each sample was then fixed in 3.7% formaldehyde for 20min, permeabilized with 0.5% Triton X-100 for 10min and blocked in 1% Bovine Serum Albumin for 60min, all at room temperature. Each sample was incubated with its specific primary antibodies or Alexa Fluor 594-phalloidin (1:100 dilution, A12381, Thermo Fischer Scientific) overnight at 2–8°C. The following primary antibodies were used: anti-CD90 (1:50 dilution, ab133350, Abcam); anti-E-cadherin (1:100 dilution, ab40772, Abcam); anti-EGFR (1:100 dilution, AHR5072, Thermo Fisher Scientific); anti N-cadherin (1:100 dilution, ab12221, Abcam); anti-α-tubulin (1:100 dilution, T6074 Sigma-Aldrich); anti-vimentin (1:100 dilution, V6630, Sigma-Aldrich). After three washes with PBS buffer for 10min, secondary antibodies (1:400 dilution) labelled with specific dyes were incubated according to the nature of the primary antibodies used. Nuclei were stained with 4′,6-diamidino-2-phenylindole (DAPI) for 10min. Finally, the slides were mounted with Vectashield mounting media and the immunostained coverslips were examined under a confocal laser scanning microscope LSM-510-Meta (Carl Zeiss). The stack images of 100 optical sections with a step size of 200 nm were then deconvolved in three dimensions with the AxioVision 4.1 constrained Iterative Algorithm (Carl Zeiss), using the ImageJ software for the analysis.

#### Colony formation assay in semi-solid medium

Each well of a 24-well plate was coated with 500μL 0.6% agarose (Fisher Scientific) solution in specific medium for each lineage. After agarose polymerization 10^3^, 10^4^ and 10^5^ cells/well were then seeded on top of the 0.6% agarose and allowed to stand for about 10min before the addition of 500μL of melted 0.3% agarose in specific medium for each lineage. After agarose polymerization, 500 μL of specific medium for each lineage were added. The liquid medium was changed every two days. After 14 and 21 days, cells were fixed in 3.7% formaldehyde and colonies were counted under the EVOS Fl Fluorescence imager (Life Technologies).

#### Cell invasion and migration assays

The cell invasion and migration assays were performed as described by Trombetta-Lima et al. [19]. In brief, 1 × 10^5^ cells were seeded into each chamber of a 12-well micropore membrane filter with 8μm pores (Becton Dickinson Labware) for the migration assay or seeded into the chamber of a 12-well Matrigel-coated membrane filter (Becton Dickinson Labware) for the invasion assay. The bottom chamber was filled with DMEM containing 10% FCS for Hs578T cell lines and DMEM/F12 supplemented as aforementioned for MCF10A cell lines, as chemoattractants. After 24-48h incubation at 37°C, the membranes were fixed and stained with 0.125% Coomassie Blue 250-R in methanol: acetic acid: H_2_O (45:10:45, v/v/v) for 2min. All of the cells invading through the membrane were counted under an inverted light microscope at 100X magnification.

#### Luciferase reporter assays

The AP-1 response element (PPRE) firefly luciferase reporter pGL4.44[*luc2P/*AP1 RE/Hygro] construct was introduced into both the parental MCF10A cell line and the CD90^+^ cell line derivative from it (MCF10A/CD90^+^) by transfection, using Lipofectamin 2000 (Invitrogen). The pGL3 and pRL-TK vectors were used as controls. After 5h transfection, the medium was changed to fresh medium without FCS and then supplemented (or not) with EGF (20ng/mL) for 24h. Luciferase activity was then measured using the Dual-Glo Luciferase Assay System (Promega) and read on a SpectraMax^*®*^ Paradigm Multi-Molde Microplate Detection Platform (Molecular Devices, California, USA. The luciferase readings were normalized to the control, vehicle only-treated samples, for statistical analysis.

#### *In vivo* tumorigenicity studies

To evaluate the metastatic potential of each cell lineage, 1 × 10^6^ cells were injected directly into the tail vein of 14 to 18-week-old male immune deficient Rowett Rats (NTacFCfiq: NIJ-Whn). After 45 days, rats were euthanized, and the lungs were removed and fixed in 3.7% formaldehyde for colony couting. This study was in agreement with the Ethical Principles in Animal Research adopted by the Brazilian College of Animal Experimentation (COBEA) and has benn approved by the Internal Animal Care and Use Committee of the Institute of Chemistry, University of São Paulo Chemistry Institute on 02/16/2011. Two independent experiments were performed.

#### CD90 immunohistochemistry

The lungs of the rats were analyzed by immunohistochemistry (IHC) using CD90 antibody (Abcam, ab133350). The formalin fixed paraffin-embedded (FFPE) lungs were sectioned into 3-5μm-thick sections using a microtome and mounted on polylysine-treated slides. The IHC assay was performed by a service facility. Heat-mediated antigen retrieval was performed at 97°C for 20 min, transferred to 65°C and washed in PBS. To block endogenous peroxidase, the tissues were incubated with hydrogen peroxide for 10min at room temperature, washed in in PBS for 5 min. After blocking of non-specific sites, the tissues were incubated for 40 min with polyclonal antibody against CD90 (1:200 dilution) at Dako Flex solution, and the slides were washed two times in PBS for 5 min. The slides were incubated with rabbit secondary antibody for 15 min and washed again. Then, tissues were incubated for 15min with the biotinylated link and with streptavidin conjugated to horseradish peroxidase for 20 min. Tissues were revealed using the DAB kit (Dako North America) and counterstained with Harris hematoxylin. The sections were washed in water, dehydrated in Ethanol for 30 seconds (70%, 95%, 100%), xylene embedded and assembled with Entellan® new mounting medium (Merck).

#### Statistical analysis

Statistical analyses were performed using the GraphPad Prism 5.0 program. ANOVA test followed by Tukey’s test for post hoc comparison were used for statistical analysis. Statistically significant differences were considered when p<0.05.

For TMA all analyses were carried out using the Graph Pad Prism 5.0 (Graph Pad Software Inc., USA), Mini-Tab software (v.16), and statistical software R (http://www.r-project.org). To identify the statistically significant associations between CD90 H-Score level and several clinical parameters such as age at diagnosis, tumor size, node involvement, histological grade, ER, PR, HER2, and pAkt status, we used the Chi-square test. Overall survival rates were evaluated using the nonparametric Kaplan-Meier survival curves. The Log-Rank test was used to compare the survival distributions. Cox proportional hazards regression, including several covariates, such as histological grade, tumor size, age at diagnosis, ER/PR status, and adjuvant therapy were used to estimate the adjusted hazard ratios and their statistical significance. A p-value of less than 0.05 was considered statistically significant.

## Results

### CD90 expression profile in breast cancer tissues

In order to assess the expression of CD90 in human breast cancer samples, we first used immunohistochemistry reactions in TMA slides, each containing ~300 fragments of tissues from a cohort of patients from the AC Camargo Cancer Hospital (São Paulo, Brazil) (S1 Table).

**Fig 1:**
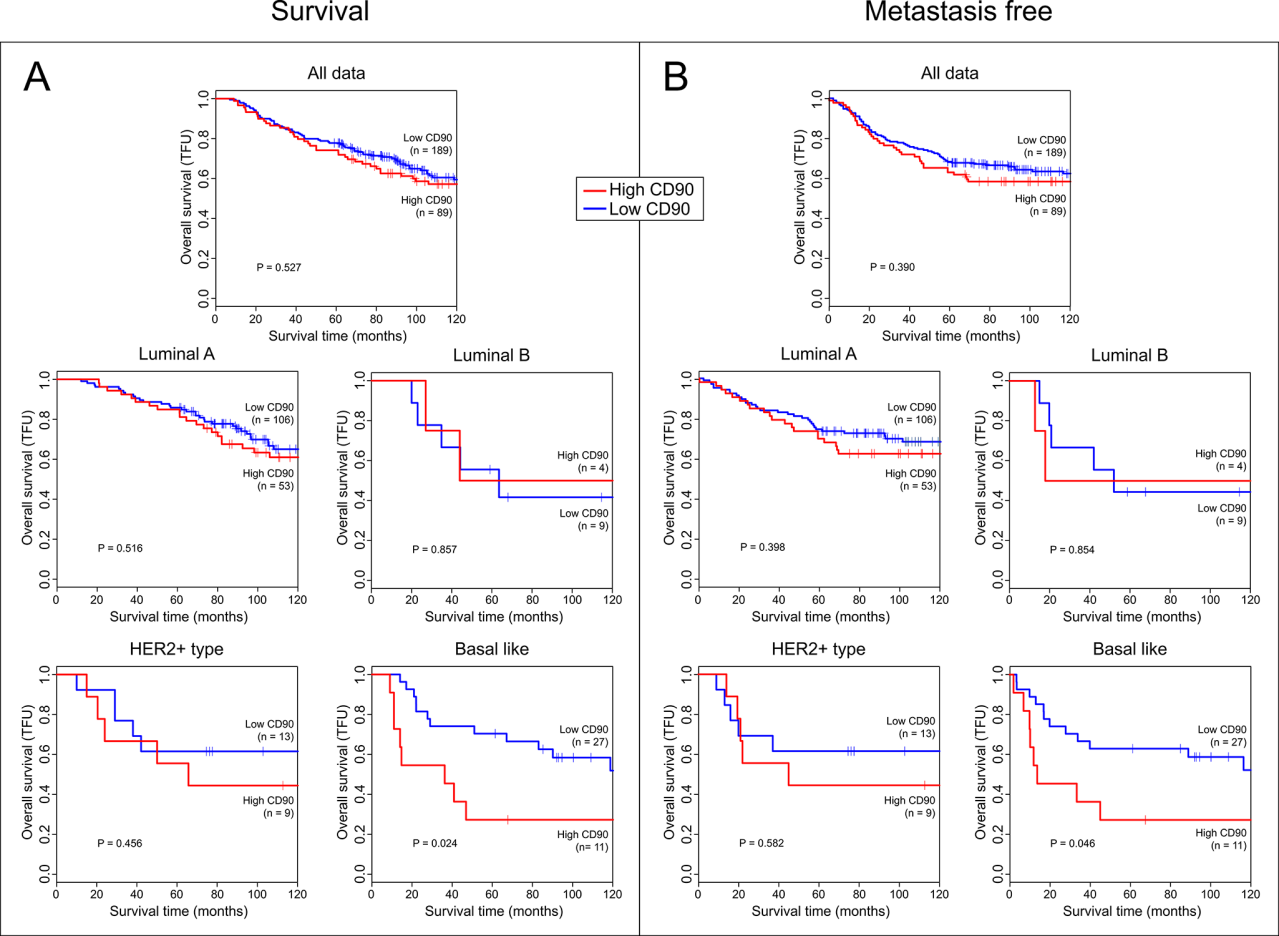
Kaplan-Meier survival analysis. (A) Kaplan-Meier (KM) survival curves at the 120-month follow up point (TFU). (B) Kaplan-Meier metastasis-free survival (MFS) curves at the 120-month follow up point. For both TFU and MFS KM survival curves, the median of the CD90 H-score was used to discriminate patients with high CD90 expression (red line) and low CD90 expression (blue line). P-values were obtained by log-rank tests.

The Kaplan Meyer curves for CD90 expression (Fig 1A and 1B) were plotted considering only the tumor areas, as described above. Considering the IHC staining in tissue samples of the patient’s cohort, we observed that CD90 is expressed in several different cell types, which are present in the tumor (S1 Fig). The stromal areas surrounding the tumors were substracted using the ImageScope Software **(**Aperio systems inc.), following the measurement evaluation method described by Zarrella et al [20] (S2 Fig). CD90 positivity of the total area of the TMA spot (S3 Fig and S2 Table) was used to generate the Kaplan Meyer curves for survival and metastasis-free survival (Fig 1A and 1B).

### Relationship between CD90 expression and other variables

CD90 positivity was classified as “high” and “low” with respect to the mean CD90 positivity value, i.e., patients with positivity higher than the mean were considered as “high” and otherwise, as “low”. The distribution of demographic, clinical, and pathological variables among CD90 “low” and “high tumors” are shown in S3 Table. CD90 “high” positivity patients did not significantly differ from CD90 “low” positivity patients for all evaluated characteristics, namely: age at diagnosis (p = 0.119), tumor size (p = 0.959), Perou/Solie classification (p = 0.638), lymph node status (p = 0.859), histological grade (0.411), adjuvant therapy (p = 0.538), ER (p = 0.738), PR (p = 0.557), and HER2 (p = 0.717) status. In a separate analysis (Kaplan-Meyer curves of Fig 1 and Cox Regression analysis in S4 and S5 Tables, respectively), we found a positive correlation between “high” CD90 expression with high Mortality rate in TFU (p = 0.032), but only for the basal-like tumors.

CD90 positivity is an independent prognosis factor of TFU in the basal-like subgroup (hazard ratio HR = 1.78, 95% confidence interval (CI) = 1.05–3.01, p = 0.032) (S3 Table), therefore, high CD90 positivity in basal-like tumors is an indication of worse prognosis. For the remaining subgroups (Luminal A, Luminal B and Her2), there was no statistical evidence to state that CD90 is independently associated with prognosis. The TMA data are shown at S5 Table and images are available at https://figshare.com/s/4512a0c24a9b295cdf75.

### Morphology and doubling time

**Fig 2:**
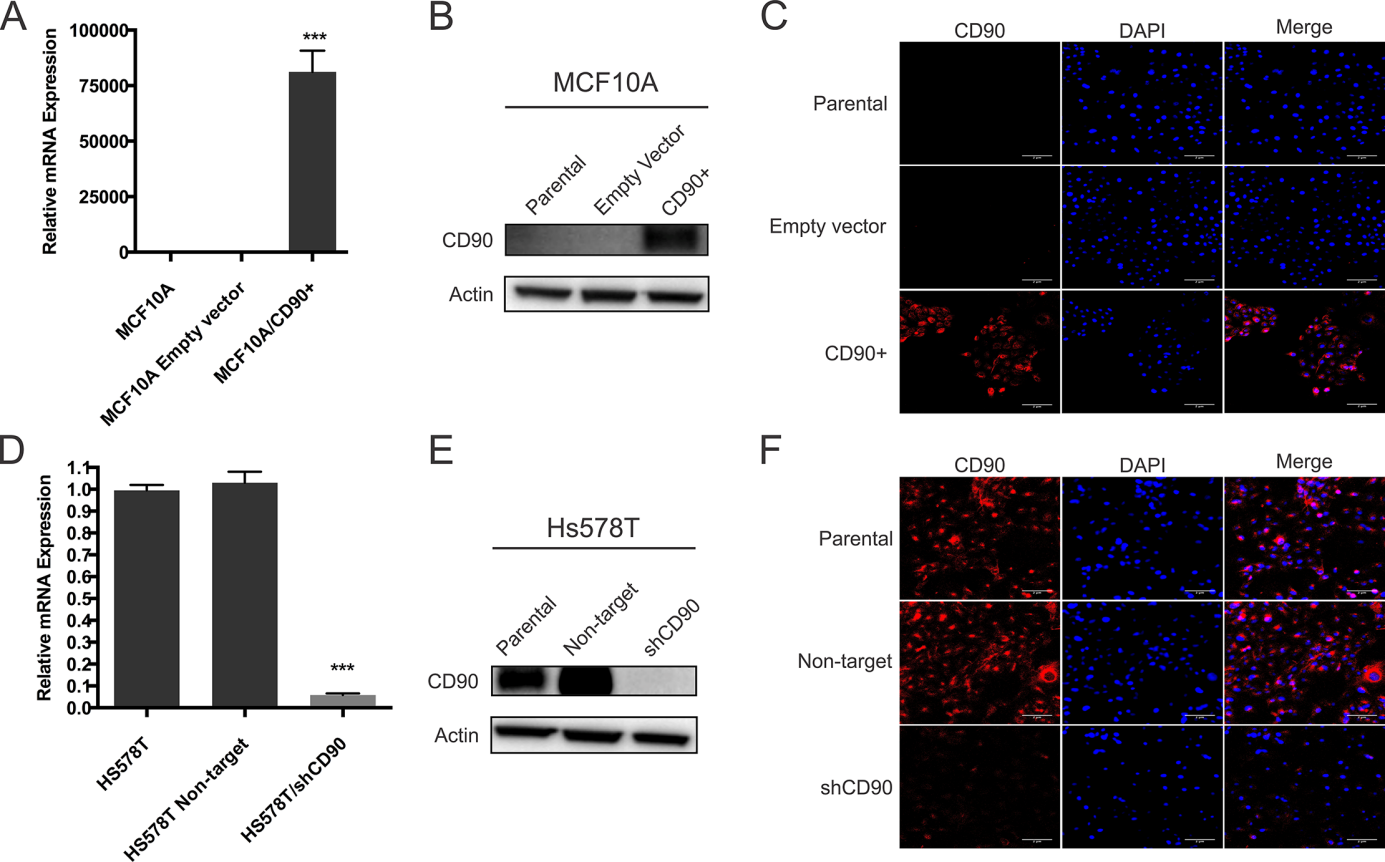
Characterization of CD90 expression in MCF10A and Hs578T and transformed cells. The expression of CD90 was analysed by qRT-PCR, Western-blot and immunofluorescence microscopy in MCF10A (A,B,C) and Hs578T (D,E,F) cell lines. For Western blot analyses, the cells were lysed and the total protein was separated by SDS-PAGE. The gels were electrotransfered onto nitrocellulose membranes. The membranes were then stripped and reprobed with β-actin antibodies to confirm that equal amounts of each protein extract were loaded. The immunoblots shown are representative of a total of three independent experiments. CD90 (red), DAPI (blue), and merged images (original magnification, x20).

**Fig 3:**
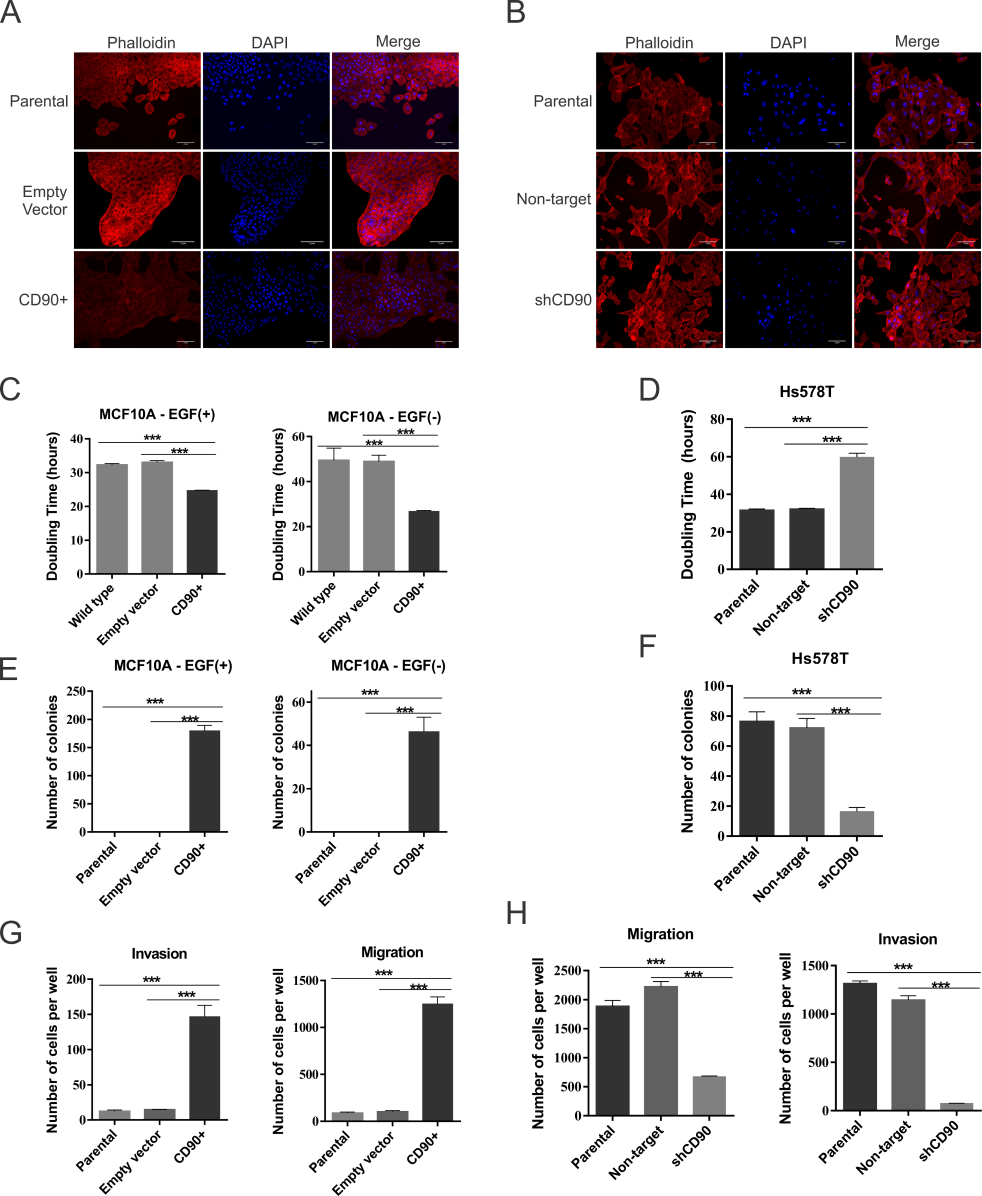
MCF10A, Hs578T and transformed cells characterization. (A, B) Cell Morphology analysis with phalloidin; (C, D) *In vitro* cell growth analysis; (E, F) Colony formation assay in semi-solid medium; (G, H) Migration and invasiveness assays. ANOVA test followed by Tukey’s test for post hoc comparison were used for statistical analysis. The results are presentes as mean±SD. Asterisks show statistically significant expression differences (p <0.05). The experiments were performed in three independent experiments. Phalloidin (red), DAPI (blue), and merged images (original magnification, x20).

Upon confirming CD90 overexpression in MCF10A cells (Fig 2A–2C) and knockdown of this gene in the Hs578T cells (Fig 2D–2F), we assessed the morphological and growth characteristics of these cells, when compared with their respective parental cell lines and empty vector or non-target constructs (Fig 3A–3D). Fig 3A and 3B show the immunofluorescence results for F-Actin based on Phalloidin staining, for both lineages (MCF10A and Hs578T). The results indicate that overexpression of CD90 in MCF10A cells leads to change in the organization of the actin filaments (Fig 3A), whereas the CD90 knockdown in Hs578T cells leads to a slight decrease in cell size but did not demonstrated a significant difference in the actin staining (Fig 3B). To better assess the cytoskeleton structure in our cell lines, we used immunofluorescence for tubulin in the MCF10A and Hs578T cell lineages, however, the results did not indicate a clear modification of the microtubules, as shown in S4 Fig and S5 Fig, respectively.

Normally, MCF10A cells are epidermal growth factor (EGF)-dependent for growth. However, when overexpressing CD90 (MCF10A/CD90^+^), the cells present a marked difference in cell growth, becoming EGF-independent, as may be judged from their doubling time (DT) difference (Fig 3C). MCF10A/CD90^+^ cells display a higher growth rate, when compared to parental and empty vector cell lines, both in the presence and in the absence of EGF (Fig 3C). Regardless of EGF supplementation, the doubling time of MCF10A/CD90^+^ cells (approximately 20h) was much lower than that of the control parental and empty vector cell lines, which reached 50h in the absence of EGF (Fig 3C).

It is noteworthy that the genetically modified Hs578T/shCD90 lineage displays a slower growth rate (DT = 60h), when compared to the parental cells (DT = 32h) or with cells transduced with the control non-target vector (DT = 32h). From this analysis, it was observed that the Hs578T/shCD90 cells have twice the doubling time, when compared to the parental cells and to cells transduced with the control vector (Fig 3D), thus confirming the slower growth rate of the cells in which CD90 had been silenced.

### CD90 overexpression renders normal MCF10A cells anchorage-independent

Since the aforementioned studies indicated that CD90 overexpression leads to cell transformation, we assessed the effect of CD90 overexpression on growth in semi-solid medium (agarose). Moreover, due to the importance of EGF for growth of the MCF10A cell line, the tests with this line and its derivatives were also carried out in the presence and absence of this peptide growth factor.

Fig 3E shows that the parental MCF10A and MCF10A-empty vector-transduced cells were unable to form colonies in agarose (S6A Fig), irrespective of the presence of EGF in the medium. On the other hand, the MCF10A/CD90^+^ cells, which overexpress CD90, displayed the ability to form colonies in the absence of EGF, but the number of colonies was greater when this growth factor was added to the medium (Fig 3E), indicating the anchorage-independence of this lineage. As expected, both parental Hs578T and non-target transduced Hs578T cells were capable of forming colonies in agarose (Fig 3F and S6B Fig). However, as shown in Fig 3F, for the Hs578T/shCD90 cell line, the total number of colonies and the number of large colonies were significantly decreased and the vast majority of the colonies were small, when compared to the control lines (Fig 3F and S6B Fig), indicating that CD90 knockdown clearly compromised the ability of Hs578T cells to grow in semi-solid medium.

### Role of CD90 in breast cancer cells invasiveness and migration

The invasive and migration potential of the MCF10A and Hs578T cell lines and of their genetically modified derivatives were analyzed. Fig 3G shows that among the MCF10A cell lineages, only the MCF10A/CD90^+^ lineage was capable of migration and invasion. On the other hand, for the MCF10A-parental and MCF10A-empty vector cell lines only an insignificant number of cells was found in the lower chamber of the wells.

The corollary experiment, shown in Fig 3H, shows that CD90 silencing in the Hs578T/shCD90 cells, led to a significant decrease in the number of cells which were able to migrate to the lower chamber of the transwell and, also, to a low invasiveness through the pores clogged with the Matrigel^TM^ basal membrane, in comparison with the controls (parental Hs578T and non-target vector), which were both able to migrate and invade the coated plate towards the chemoattractant agent.

### Analysis of the EGF signaling pathway

**Fig 4:**
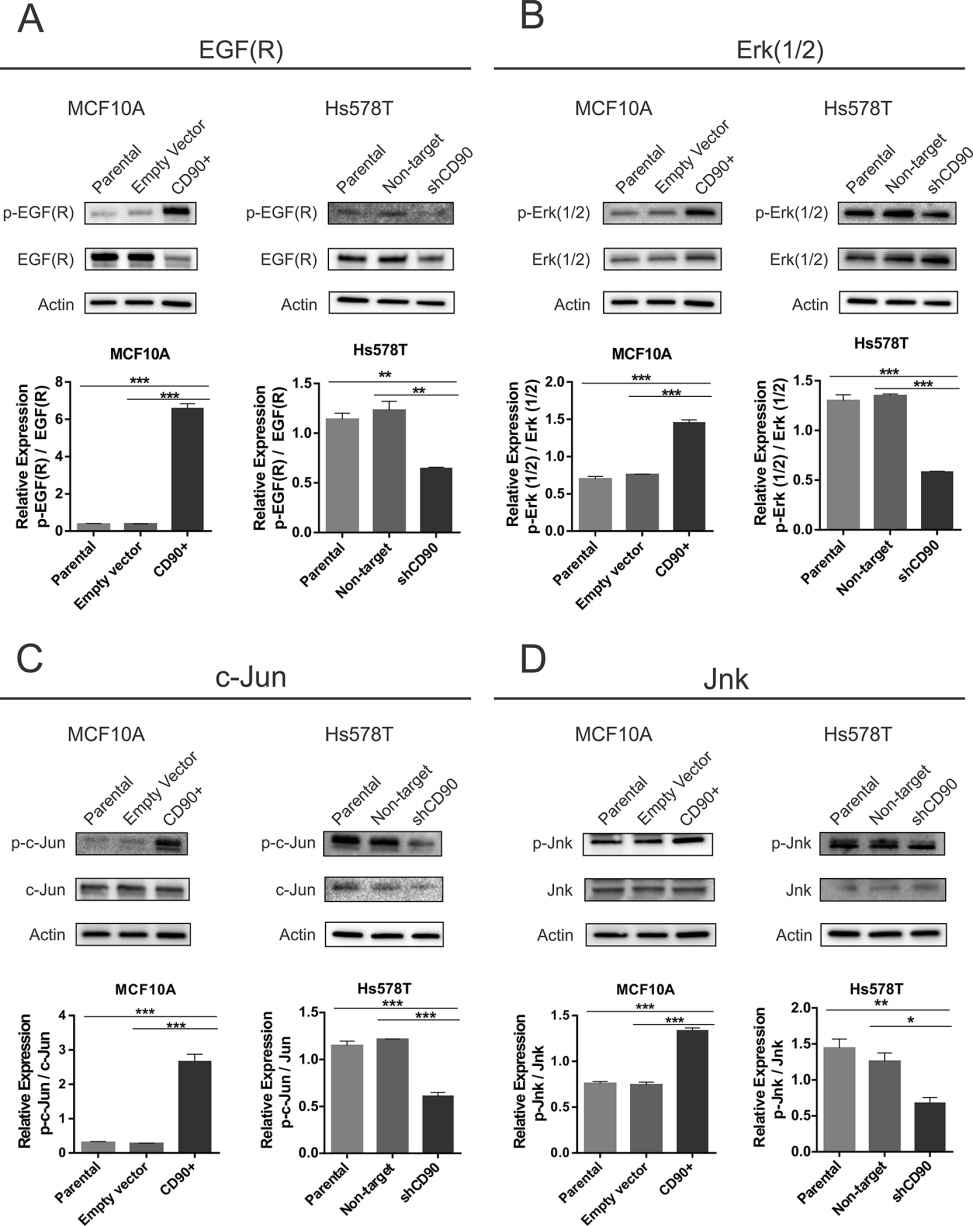
Analysis of EGF signaling. The cells were lysed and the p-EGF(R)/EGF(R) (A), p-ERK1/2/ERK1/2 (B), p-cJun/cJun (C), p-JNK/JNK (D) were analysed by Western blot. The membranes were stripped and reprobed with a β-actin antibody to confirm that equal amounts of each protein extract were loaded. The immunoblots shown are representative of a total of three independent experiments. The corresponding histograms of the figure show arbitrary units of normalized densitometric values. ANOVA test followed by Tukey’s test for post hoc comparison were used for statistical analysis. The results presented as the mean ± SE of three independent experiments (p <0.05).

Since we found that, contrary to the parental MCF10A line, the MCF10A/CD90^+^ cells were able to grow independently of EGF (Fig 3C), we decided to investigate the activation of the EGF signaling pathway, by Western blotting (Fig 4). Due to the requirement for the presence of EGF in the MCF10A culture medium, cells of this lineage and of the corresponding ones transformed were deprived of this growth factor only 48h before protein extraction. Positive immnofluorescence results were also observed for EGFR in all three MCF10A cell lines (S7 Fig). As shown in Fig 4A, significantly increased levels of EGF receptor phosphorylation were found for the MCF10A/CD90^+^ lineage, when compared with the parental (MCF10A) and control cells transformed with the empty vector MCF10A cells. Significant reduction in the phosphorylation levels of the EGF receptor were observed for the Hs578T/shCD90, when compared with the parental Hs578T cells and with those cells transformed with the non-target vector (Hs578T non-target). The same pattern was observed for Erk (1/2) proteins (Fig 4B), c-Jun (Fig 4C) and Jnk (Fig 4D), in which the phosphorylated levels were higher in cells expressing CD90 (MCF10A/CD90^+^, Hs578T, Hs578T non-target) and reduced in cells which do not express CD90 (MCF10A parental and MCF10A empty vector) or display lower CD90 expression (Hs578T/shCD90).

**Fig 5:**
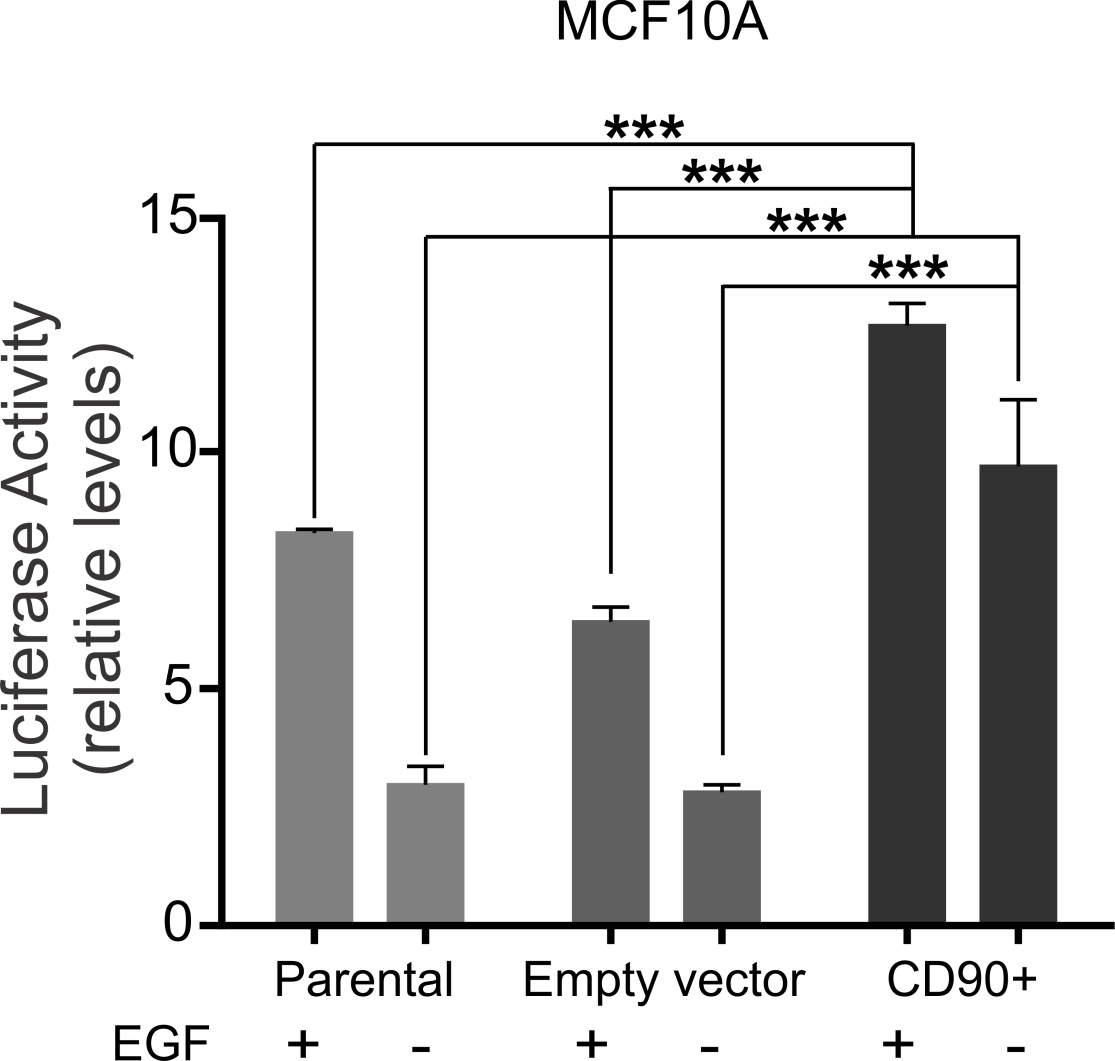
Analysis of the activity of the responsive element of the transcription factor AP-1. MCF10A, MCF10A-empty vector and MCF10A/CD90+ cells (2x10^5^) were plated and transfected with vector pGL4.44 and pRL-TK, deprived for FCS and EGF or treated with EGF. The reading of luciferase activity was done after 48 hours of transfection. The luciferase activity was normalized by the activity/constitutive expression of Renilla. ANOVA test followed by Tukey’s test for post hoc comparison were used for statistical analysis. Asterisks show statistically significant expression differences (p <0.05). The experiments were performed in three independent experiments.

Since we observed that, in the absence of EGF, CD90 overexpression in the MCF10A cell line leads to induction of phosphorylated proteins which are essential for activation of the EGF signaling pathway and it is well known that this pathway results in activation of AP-1 (activating protein-1) transcription factor, we decided to assess whether the MCF10A/CD90^+^ lineage also displayed activated AP-1 in the absence of EGF (Fig 5). The results of this analysis (Fig 5) show that the MCF10A/CD90^+^ lineage displays three times higher AP1-luciferase activity when compared to the parental (MCF10A) and control empty vector MCF10A cell lineages, even in the absence of EGF treatment.

### CD90 overexpression promotes epithelial-mesenchymal transition switch

**Fig 6:**
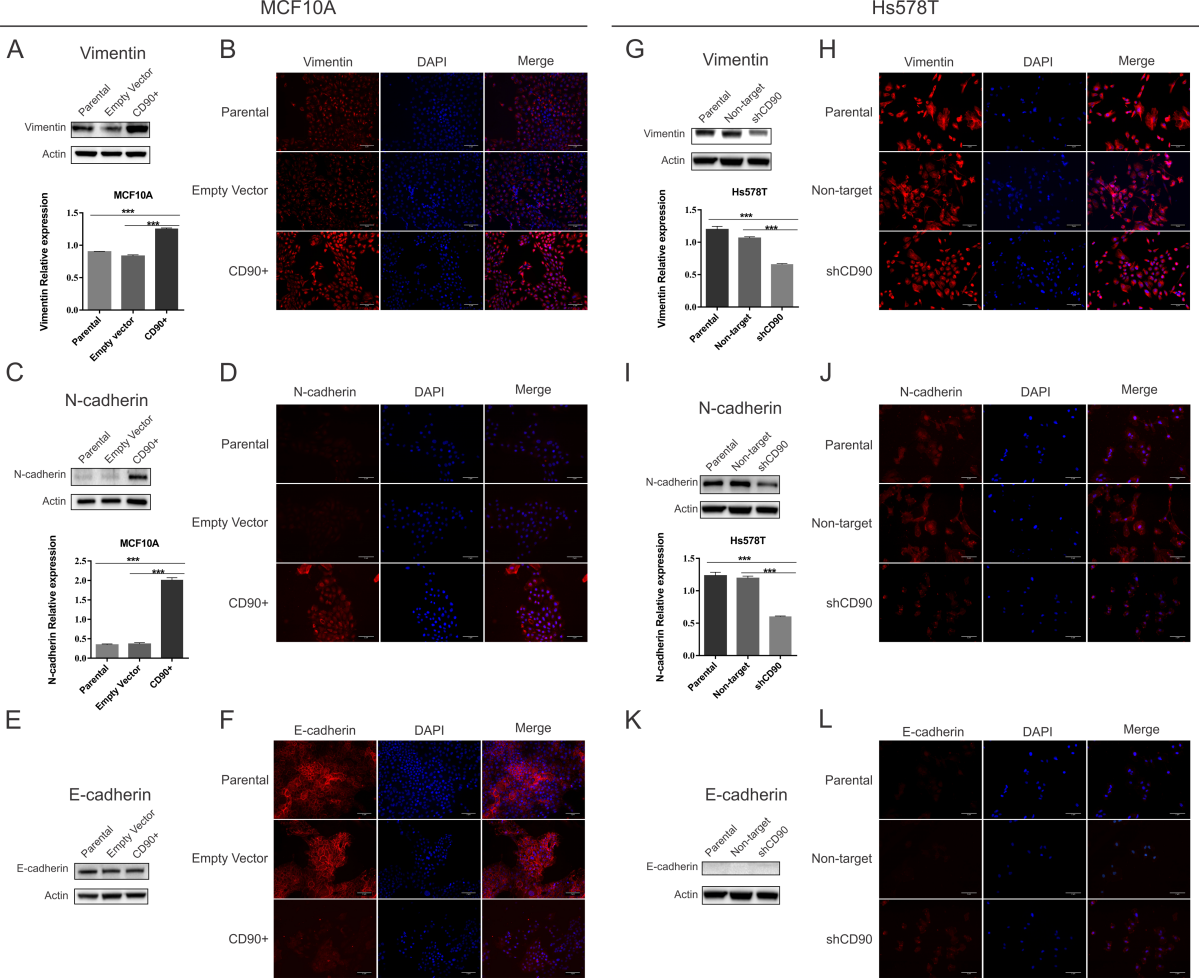
Analysis of mesenchymal and epithelial markers of MCF10A, Hs578T and transformed cells characterization. The expression of vimentin, N-cadherin, E-cadherin were analysed by Western blot (A, C, E, G, I, K), and immunofluorescence microscopy (B, D, F, H, I, L). For Western blot analyses, the cells were lysed and the total protein was separated by SDS-PAGE. The gels were electrotransfered onto nitrocellulose membranes. The membranes were then stripped and reprobed with β-actin antibodies to confirm that equal amounts of each protein extract were loaded. The immunoblots shown are representative of a total of three independent experiments. The corresponding histograms shown in the figure are expressed in arbitrary units of normalized densitometric values. ANOVA test followed by Tukey’s test for post hoc comparison were used for statistical analysis. The results are presented as the mean ± SE of three independent experiments. Asterisks show statistically significant expression differences (p <0.05). Vimentin, N-cadherin and E-cadherin (red), DAPI (blue), and merged images (original magnification, x20).

Due to the transformation potential of CD90 in the non-tumorigenic MCF10A, we investigated whether CD90 could induce epithelial/mesenchymal transition. When the mesenchymal markers were analyzed in MCF10A cells, we observed that vimentin (Fig 6A and 6B) and N-cadherin (Fig 6C and 6D) were more expressed in the MCF10A/CD90^+^ cell line. Western blot results, shown in Fig 6E, indicate that only a slight difference in E-cadherin expression was found between MCF10A/CD90^+^ and the MCF10A-parental cell line. In order to further investigate E-cadherin expression in these cell lines, we used immunofluorescence assays. The results (Fig 6F) indicate lower E-cadherin immunoreactivity, distributed throughout the cytoplasm of MCF10A/CD90^+^ cells, when compared to MCF10A and MCF10A-empty vector cells, which display E-cadherin in the plasma membrane. The expression of vimentin and N-cadherin markers was significantly reduced in the Hs578T/shCD90 lineage (vimentin Fig 6G and 6H and N-cadherin Fig 6I and 6J), when compared to the parental (Hs578T) and control (Hs578T non target) cells, which do not express E-cad, as judged by both the Western blot and immunofluorescence results (Fig 6K and 6L), whereas the CD90 knockdown cells (Hs578T/shCD90) clearly show positive staining in the cytoplasm, although the Western blot did not detect a significant difference.

### *In vivo* analysis of the metastatic potential of the cell lines

**Fig 7:**
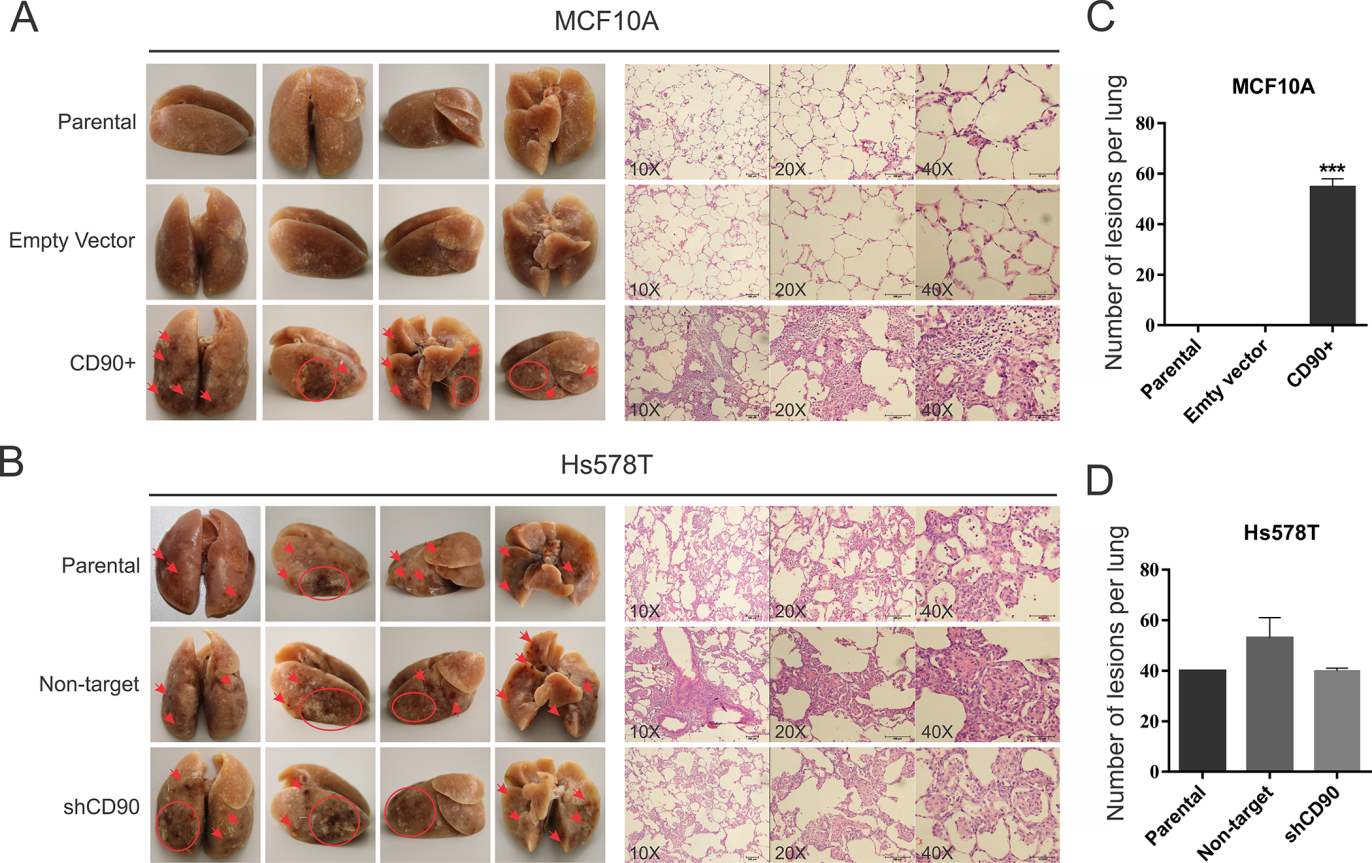
In vivo metastasis assay. *In vivo* assays for metastasis in rat lung with parental and transformed cell lines. Cells (1x10^6^) were injected into tail vein of immunodeficient rats. After 45 days the animals were euthanized and the lungs were analyzed. (A) Lungs and H&E lung sections of animals injected with the MCF10A, MCF10A-empty vector and MCF10A/CD90+ cell lines. (B) Lungs and H&E lung sections of animals injected with the Hs578T, Hs578T non-target and Hs578T/shCD90 cell lines. C) Number of lesions on the surface of the lungs of the animals injected with the cells of the strains MCF10A, MCF10A-empty vector and MCF10A/CD90+. (D) Number of lesions on the surface of the lungs of the animals injected with the cells of the strains Hs578T, Hs578T non-target and Hs578T/shCD90. ANOVA test followed by Tukey’s test for post hoc comparison were used for statistical analysis. Asterisks show statistically significant expression differences (p <0.05). The experiments were performed in two independent experiments in triplicate.

**Fig 8:**
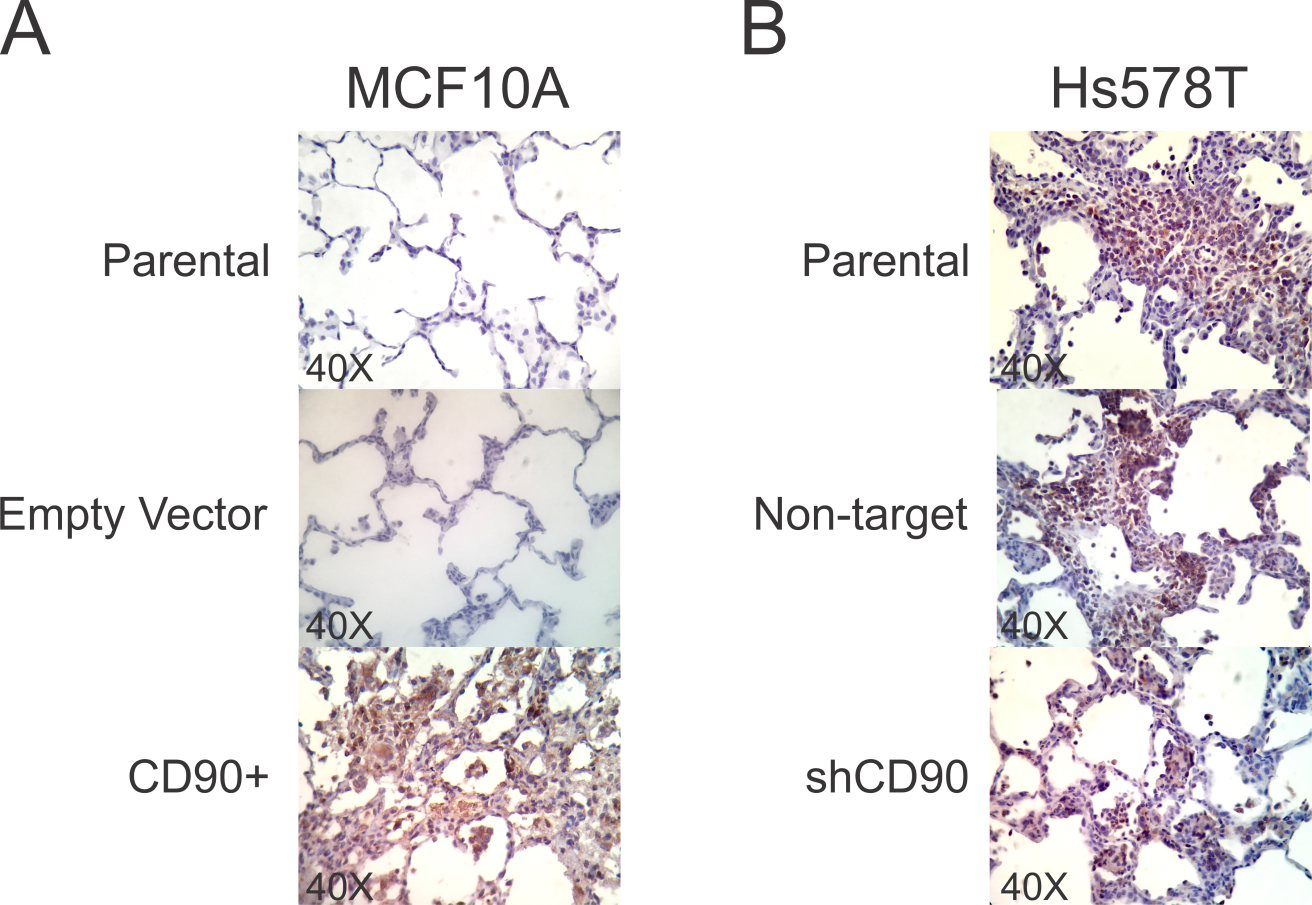
Immunohistochemistry analysis of human Anti-CD90/Thy1 of the rats lungs. Immunohistochemical staining for human Anti-CD90/Thy1 was peformed on formalin-fixed, paraffin embedded 3μm section of lungs tissues from rats injected with (A) MCF10A, MCF10A-empty vector, MCF10A/CD90+ cell lines and (B) Hs578T, Hs578T non-target and Hs578T/shCD90 cell lines. Original magnification, x40.

To investigate the role of CD90 in the metastatic potential of these breast cell lines, *in vivo* assays of experimental metastasis were carried out in immunodeficient rats, as described in Materials and Methods. After 45 days of the cells injection into the tail vein, the lungs were examined for the number of lesions (foci) appearing on the surface. The lungs of animals injected with the MCF10A/CD90^+^ cells presented dark lesions/foci on the surface, which might be regions of necrosis (Fig 7A), while this was not observed in the lungs of rats injected with the MCF10A and MCF10A-empty vector cells (Fig 7A and 7C). H&E sections of the lungs indicate that CD90 overexpression in MCF10A leads to lesions in the lung parenchyma, with syncytia formation (Fig 7A). Even though the results of MC10A cell lines demonstrated a clear difference between the CD90 overexpressing cells and the control (vector only) cells, the Hs578T/shCD90, in which CD90 was knocked down showed the same pattern, when compared to the respective parental cell line. The lungs of all rats injected with Hs578T, Hs578T non-target and Hs578T/shCD90 cells showed extensive regions of necrosis, as may be observed in Fig 7B, with no significant difference in the H&E sections (Fig 7D). We analyzed the lungs by immunohistochemistry, using an antibody specific to human CD90, identifying CD90 expression in the MCF10/CD90+, Hs578T, Hs578T/NT, Hs578T/shCD90 cell lineages, particularly in the syncytia structures present in the rats lungs (Fig 8).

## Discussion

CD90/THY-1 is a glycophosphatidylinositol-anchored cell surface protein expressed in several cell types, acting in different biological processes, such as: proliferation, cell-cell adhesion and cell migration [21]. This mesenchymal stem cell marker [22] also plays important roles in oncogenesis, being identified as a cancer stem cell marker in various malignancies, such as: glioma [23], liver [24], esophageal [25] and gastric cancer [26], being related with the poor patient outcome, metastasis and chemotherapy resistance [21]. In breast cancer, we previously reported high expression of CD90 in malignant basal-like cell lines, leading us to hypothesize that CD90 might be associated with increased malignancy grade. Here, we analyzed the expression of CD90 in human breast cancer samples and related it with the patients prognosis. For the first time, we demonstrated that high CD90 positivity is associated with metastasis and poor patient survival in the basal-like triple negative subtype (Fig 1). This result is in agreement not only with previous studies in breast cancer basal-like cell lines [9, 12], but, also, with a study which described a CD44^+^/CD90^+^ subpopulation of cells with basal-like phenotype in breast cancer samples [11]. Besides that, analysis of CD90 expression in online datasets, such as KMploter (http://kmplot.com/analysis/), demonstrated statistically significant correlations between the overall survival (OS) and relapse free survival (RFS) of breast cancer patients considering displaying high and low gene expression. These evidences corroborate our findings since the high expression of CD90 is associated at poor prognosis in Kaplan Meyers’s survival Plots.

To further explore the oncogenic role of CD90 in breast cancer, we undertook functional assays in two basal-like triple negative cell lines, the non-tumorigenic MCF10A line and the highly malignant Hs578T, by overexpressing CD90 in the former and CD90 knockdown in the latter. We observed a significant decrease in cell proliferation rate of the Hs578T/shCD90 lineage and a drastic increase in proliferation rate in the MCF10A/CD90^+^ lineage (Fig 3). An important aspect was the fact that the MCF10A/CD90^+^ lineage acquired the ability to proliferate regardless of the presence of EGF in the culture medium. Furthermore, analysis of the activity of the AP1-responsive element demonstrated that the EGF signaling pathway is also functional in the MCF10A/CD90^+^ lineage cultured in the absence of EGF (Fig 5). Even though it is still necessary to further investigate if EGFR signaling activation observed in MCF10A/CD90+ cell model is really caused by CD90 expression and not due to upstream signal or other receptors cross-talk, the possible relationship between high levels of CD90 and activation of the EGFR pathway is a promising finding in breast cancer. The expression of EGFR in breast carcinoma is associated with greater tumor size, low cell differentiation and poorer prognosis [27, 28]. More than 78% of triple negative breast cancer cases show overexpression of EGFR [29, 30]. Deregulation of the EGFR pathway by overexpression or constitutive activation of this receptor can promote tumor progression, including metastatic disease [28, 31, 32]. In this context, CD90 could be a meaningful molecule in the biology of triple negative breast cancer since its overexpression was able to increase the proliferative cell rate (Fig 3), activate the EGFR pathway (Figs 4 and 5) and, also, promote the invasion and migratory capacity *in vitro* (Fig 3) and *in vivo* (Figs 7 and 8).

The plasticity of epithelial cells constitutes a hallmark during malignant mammary carcinoma progression towards the invasive and metastatic stages. A phenomenon that could explain the distant metastases, which occur in epithelial cancers, including breast cancer, is the epithelial-mesenchymal transition (EMT) [33–35], which is characterized as loss of epithelial cell adhesion proteins, together with gain of mesenchymal-associated molecules. Our results show that both overexpression of CD90 in the MCF10A/CD90^+^ lineage and the CD90 knockdown in the Hs578T/shCD90 lineages led to a “cadherin switch” (Fig 6), a process which is usually referred to as the switch from E-cadherin to N-cadherin expression during the EMT process (M). Even though E-cadherin expression was observed in the MCF10A/CD90^+^ cell line, this expression is lower than that of the parental MCF10A lineage and, additionally, its distribution shifts from the membrane to the cytoplasm (Fig 6E and 6F). The loss of membrane E-cadherin and its translocation from the membrane to the cytoplasm and nucleus has been previously described in several metastatic cancer types [36–39]. Furthermore, it is important to note that the “cadherin switch” also includes situations in which E-cadherin expression levels do not significantly change, but N-cadherin expression is increased [40–44].

## Conclusion

Therefore, we show, for the first time, that CD90 is not only involved with malignant transformation in breast cancer cell lines, but is also correlated with metastasis and poor patient survival for the basal-like subtype, being considered as a promising new breast cancer marker to be targeted in future breast cancer therapy.

## Supporting information

S1 Fig Standardization of CD90 antibody for immunohistochemistry technique. Representative photomicrography showing Renal tissue stained by CD90 immunohistochemistry reaction (antibody diluted 1:200). Magnification: 200x.
(TIF)

S2 Fig Representative analysis of human breast cancer sample immunostained for CD90 using the ImageScope Software. (A) represents the case before the tumor area delimitation, (B) tumor area delimited usingy the “Pentool” software, (C) quantification of the pre-delimited tumor area. Magnification: 100x.
(TIF)

S3 Fig Representative photomicrography of human breast cancer stained for CD90 by immunohistochemistry. Cases were ordered in crescent order of CD90 H-score: A, 0.46, B, 17 and C, 83. Magnification: 100x.
(TIF)

S4 Fig Tubulin immunofluorescence for MCF10A cell lines. The expression of tubulin was analysed by immunofluorescence microscopy for MCF10A cell lines. Tubulin (red), DAPI (blue), and merged images (original magnification, x20).
(TIF)

S5 Fig Tubulin immunofluorescence for Hs578T cell lines. The expression of tubulin was analysed by immunofluorescence microscopy for Hs578T cell lines. Tubulin (yellow), DAPI (blue), and merged images (original magnification, x40).
(TIF)

S6 Fig Colony formation assay in semi-solid medium for MCF10A and Hs578T and transformed cell lines. Agarose cell suspension (10^4^ cells/well) were plated onto the 0.6% agarose layer in specific culture medium. 0.3% agarose was used for the top layer. After 14 days, the number of colonies was determined and photomicrographs were recorded using the EVOS Fl Fluorescence Imager Microscope, at 100x magnification.
(TIF)

S7 Fig EGFR immunofluorescence for MCF10A cell lines. The expression of EGFR was analysed by immunofluorescence microscopy for MCF10A cell lines. EGFR (red), DAPI (blue), and merged images (original magnification, x20).
(TIF)

S1 Table Cohort characteristics according clinical data. (DOC)

S2 Table Tissue microarray data. (DOCX)

S3 Table Correlation of CD90 expression with clinicopathological and molecular features of human invasive ductal carcinomas. (DOCX)

S4 Table Cox TFU correlation. (DOCX)

S5 Table Cox MFS correlation. (DOCX)
