# Supplementary figures and images for: High CD90 (THY-1) expression positively correlates with cell transformation and worse prognosis in basal-like breast cancer tumors
Source: PLoS One. 2018 Jun 27;13(6):e0199254. doi: 10.1371/journal.pone.0199254 (PMC6021101; doi:10.1371/journal.pone.0199254)

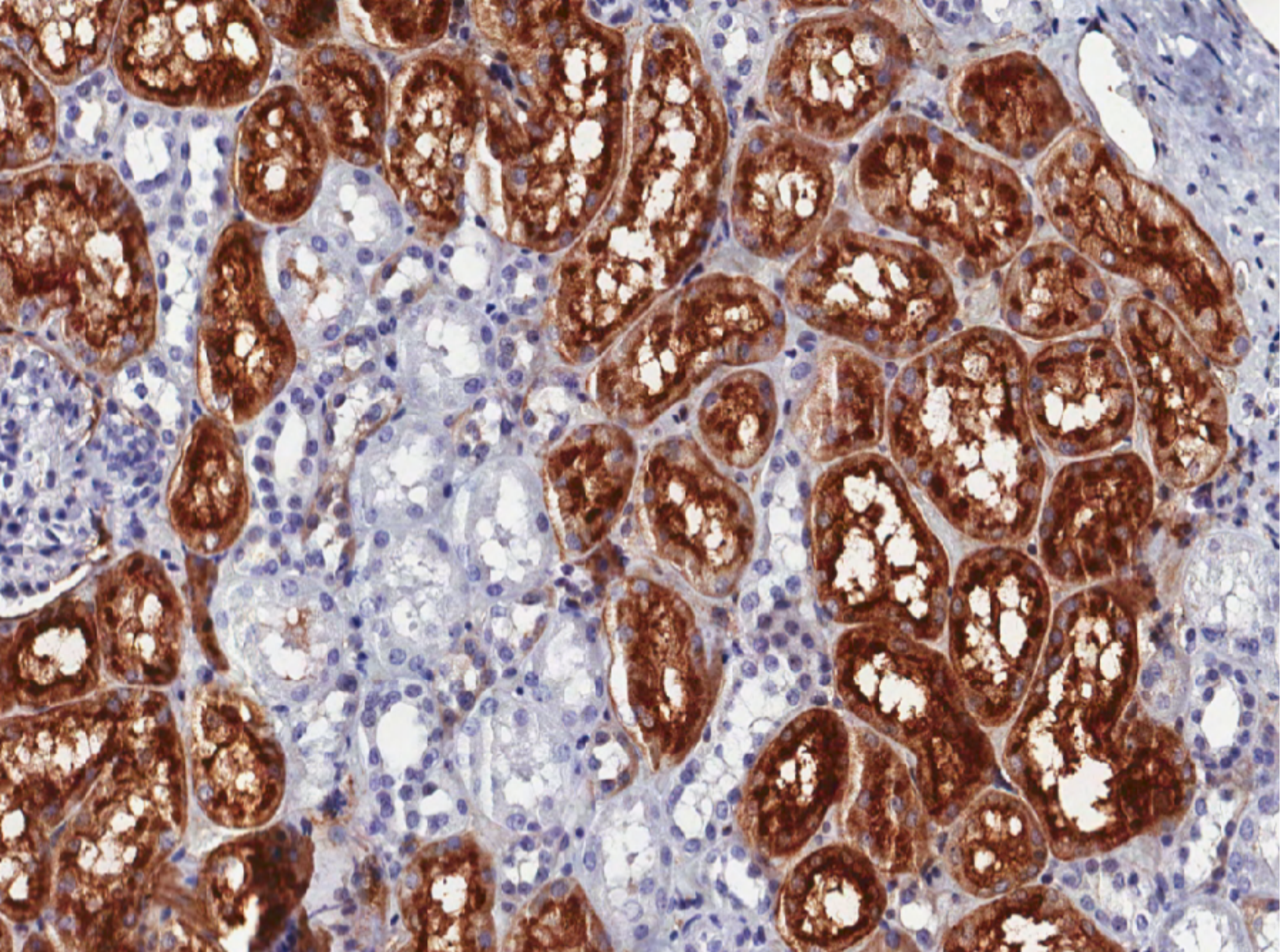

Supplement: S1 Fig — Representative photomicrography showing Renal tissue stained by CD90 immunohistochemistry reaction (antibody diluted 1:200). Magnification: 200x. (TIF) [file pone.0199254.s001.tif]

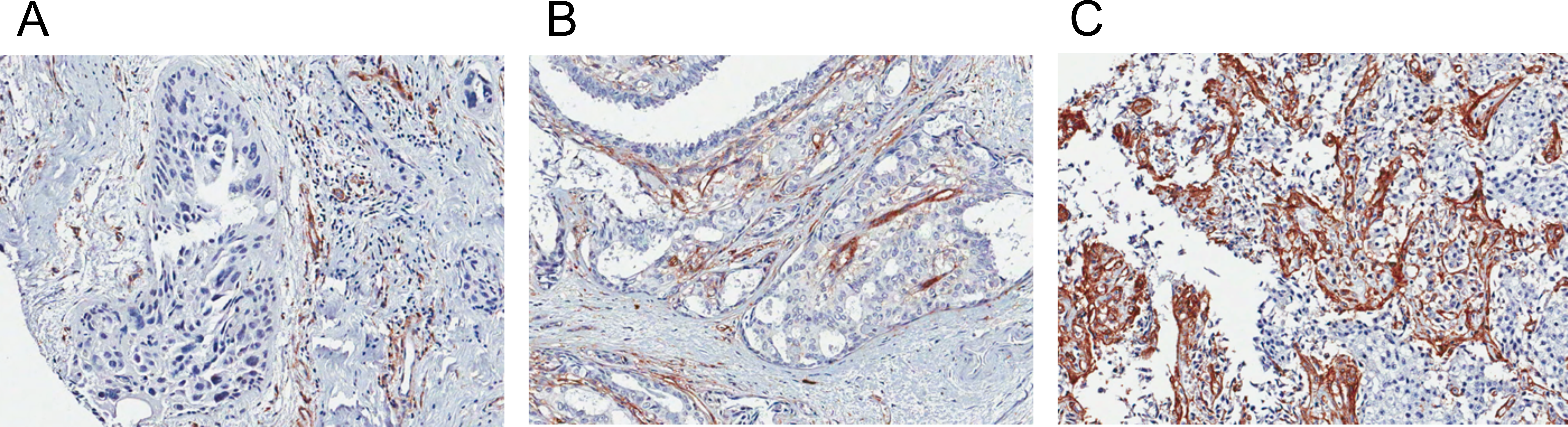

Supplement: S3 Fig — Cases were ordered in crescent order of CD90 H-score: A, 0.46, B, 17 and C, 83. Magnification: 100x. (TIF) [file pone.0199254.s003.tif]

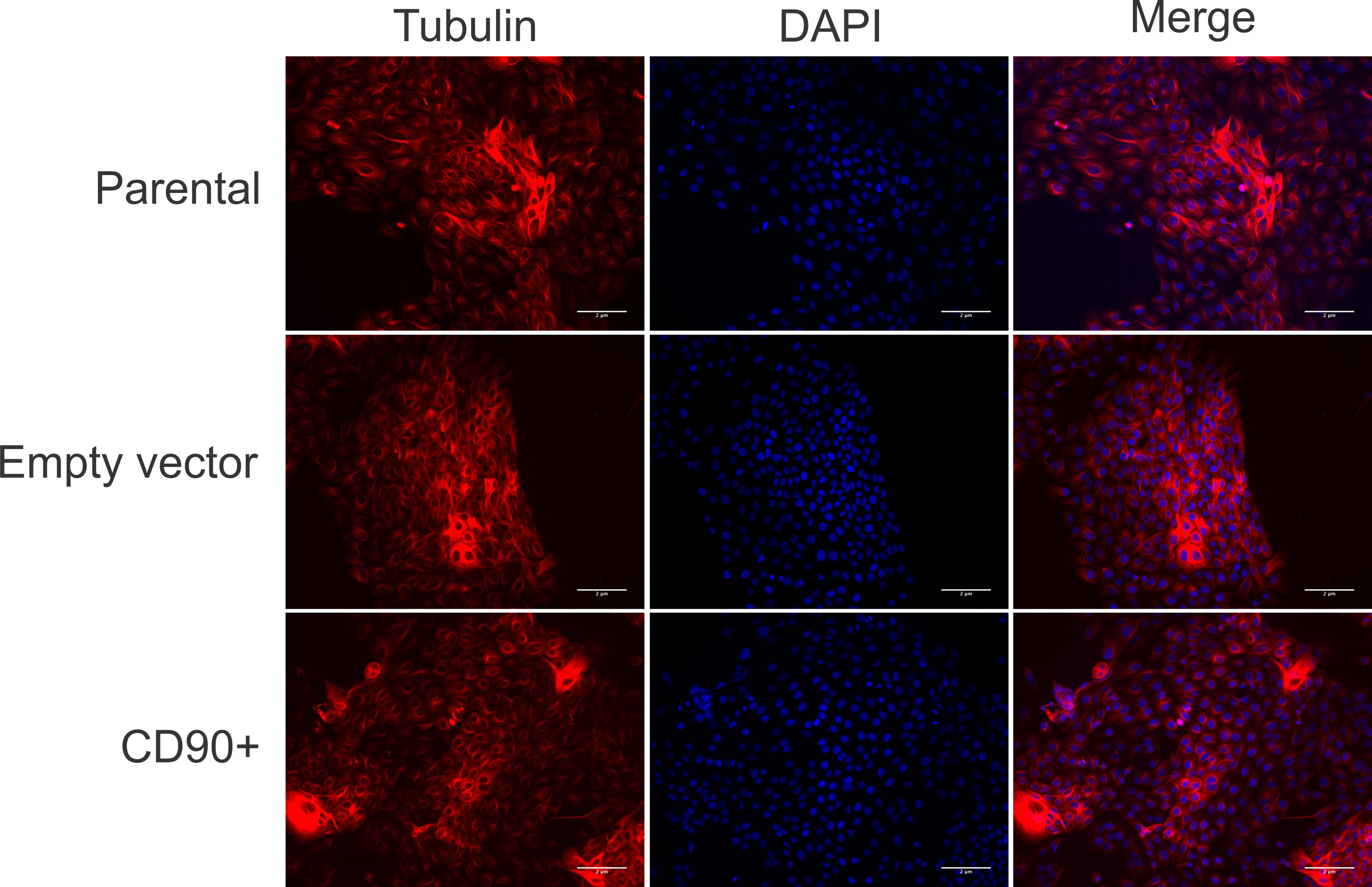

Supplement: S4 Fig — The expression of tubulin was analysed by immunofluorescence microscopy for MCF10A cell lines. Tubulin (red), DAPI (blue), and merged images (original magnification, x20). (TIF) [file pone.0199254.s004.tif]

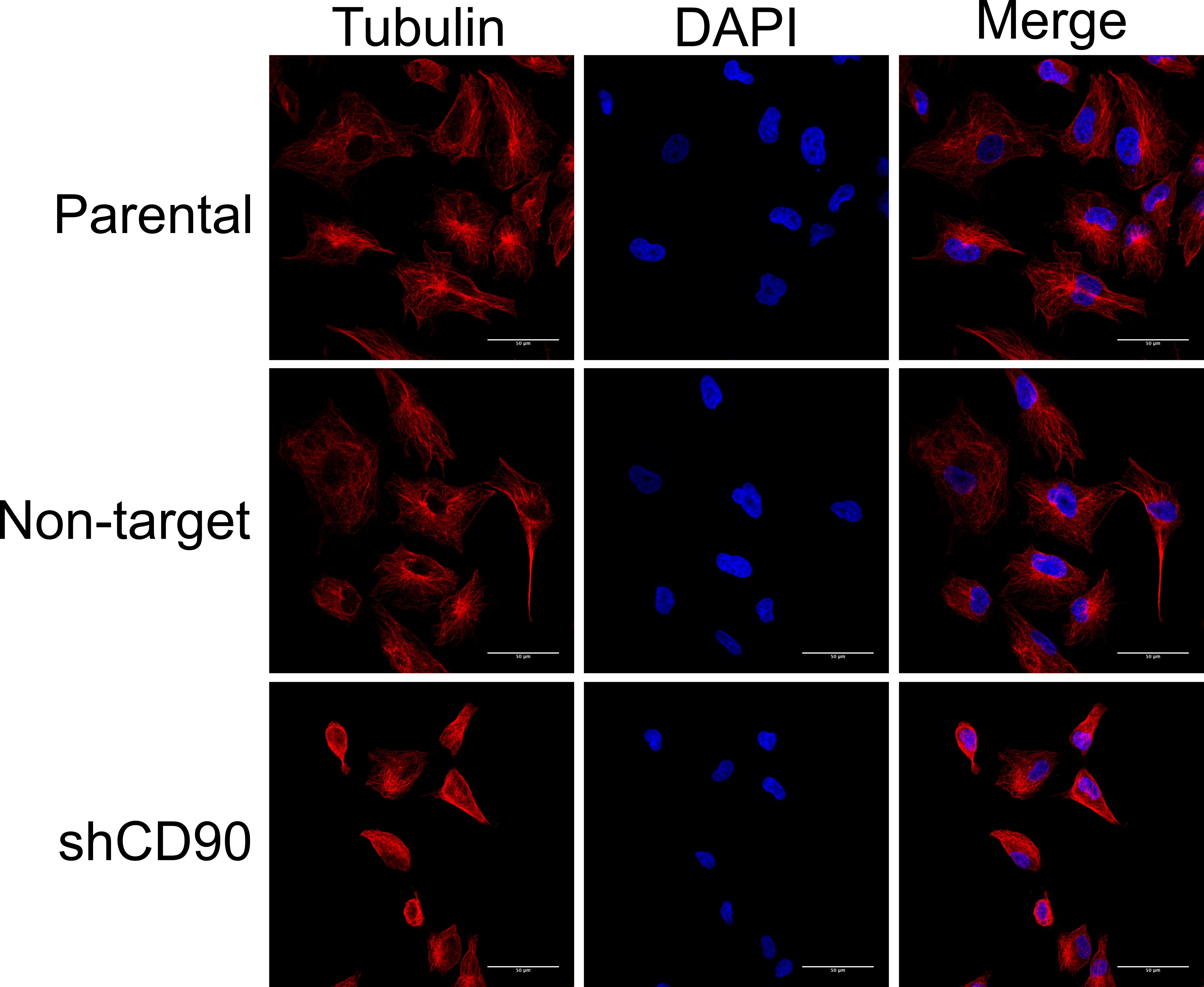

Supplement: S5 Fig — The expression of tubulin was analysed by immunofluorescence microscopy for Hs578T cell lines. Tubulin (yellow), DAPI (blue), and merged images (original magnification, x40). (TIF) [file pone.0199254.s005.tif]

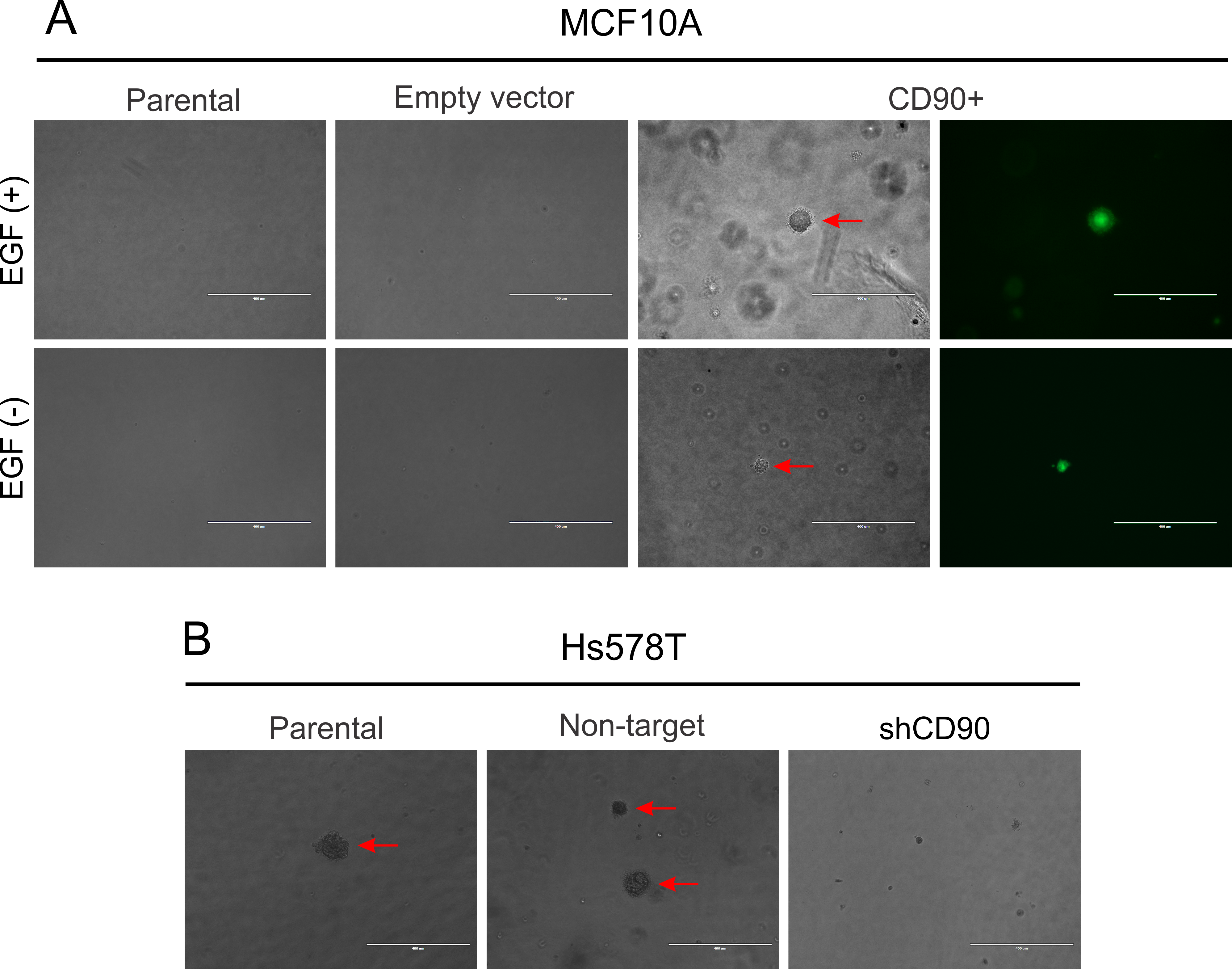

Supplement: S6 Fig — Agarose cell suspension (104 cells/well) were plated onto the 0.6% agarose layer in specific culture medium. 0.3% agarose was used for the top layer. After 14 days, the number of colonies was determined and photomicrographs were recorded using the EVOS Fl Fluorescence Imager Microscope, at 100x magnification. (TIF) [file pone.0199254.s006.tif]

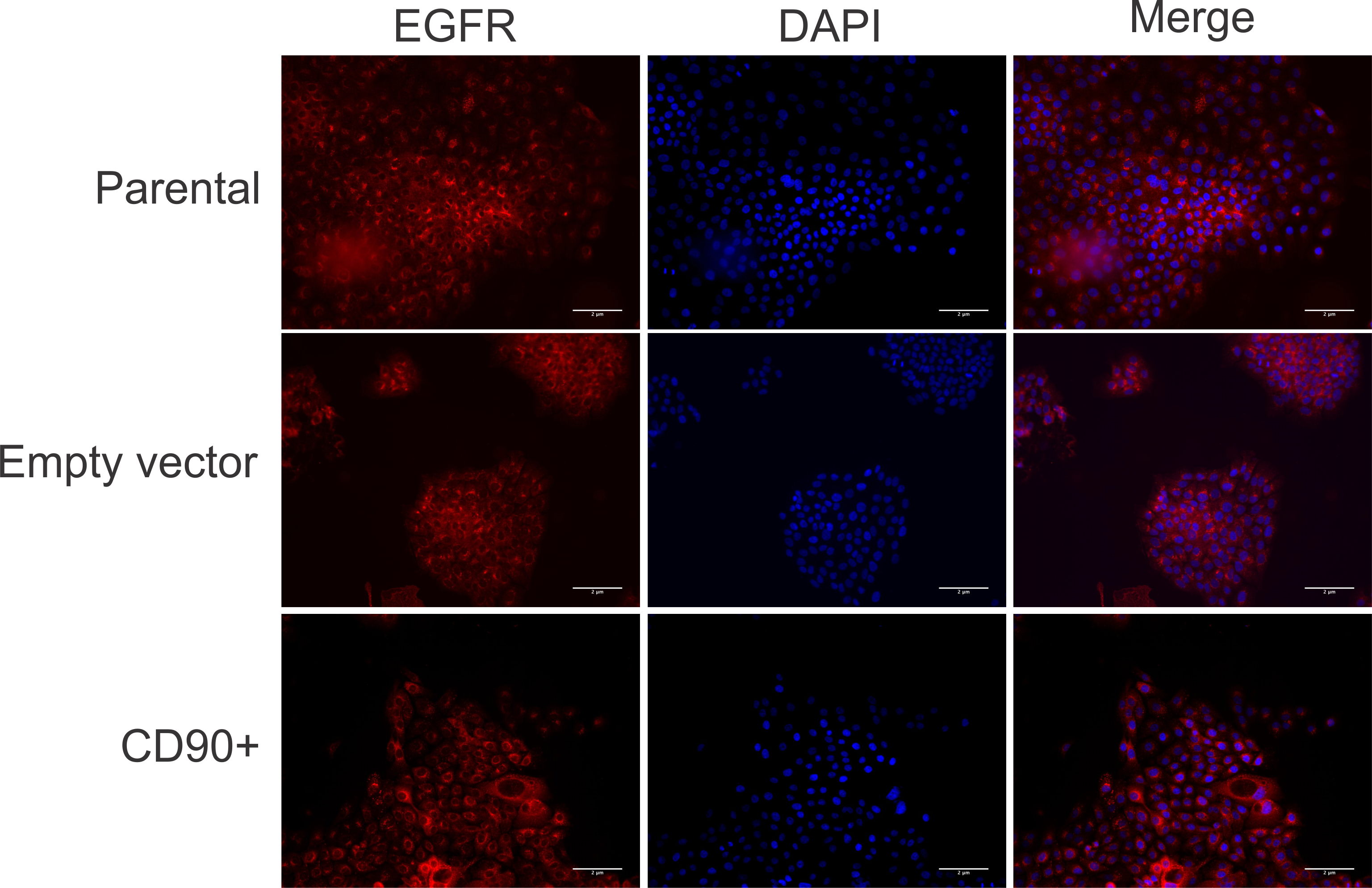

Supplement: S7 Fig — The expression of EGFR was analysed by immunofluorescence microscopy for MCF10A cell lines. EGFR (red), DAPI (blue), and merged images (original magnification, x20). (TIF) [file pone.0199254.s007.tif]
