# Supplementary material for: High CD90 (THY-1) expression positively correlates with cell transformation and worse prognosis in basal-like breast cancer tumors
Source: PLoS One. 2018 Jun 27;13(6):e0199254. doi: 10.1371/journal.pone.0199254 (PMC6021101; doi:10.1371/journal.pone.0199254)
Supplement: S1 Table — (DOC) [file pone.0199254.s008.doc]

**Supporting information**

Table S1. Cohort characteristics according clinical data.

| **Parameter** | **N** | **Median (minimum - maximum) / Percentage of cases** |
| --- | --- | --- |
|  |  |  |
| Age at diagnosis | 278 | 55 years (24 - 92) |
|  |  |  |
| Tumor size | 267 | 4.2 cm (0.8 - 14.0) |
|  |  |  |
| Tumor grade |  |  |
| 1 | 42 | 15.16 |
| 2 | 165 | 59.57 |
| 3 | 70 | 25.27 |
|  |  |  |
| Lymph node involvement |  |  |
| N0 | 95 | 34.67 |
| N1 | 89 | 32.48 |
| N2 | 54 | 19.71 |
| N3 | 36 | 13.14 |
|  |  |  |
| Molecular subtype * |  |  |
| Luminal A | 159 | 68.54 |
| Luminal B | 13 | 5.60 |
| HER2+ type | 22 | 9.48 |
| Basal like | 38 | 16.38 |
|  |  |  |
| Adjuvant therapy |  |  |
| None | 43 | 15.47 |
| Chemotherapy | 235 | 84.53 |
|  |  |  |
| Survival status |  |  |
| Alive | 154 | 55.40 |
| Dead | 124 | 44.60 |
|  |  |  |
| Follow-up |  |  |
| TFU | 278 | 98.16 months (7.00 - 240) |
| MFS | 278 | 87.36 months (0 - 240) |
|  |  |  |

* Six individuals did not present subgroup classification
