# Supplementary material for: High CD90 (THY-1) expression positively correlates with cell transformation and worse prognosis in basal-like breast cancer tumors
Source: PLoS One. 2018 Jun 27;13(6):e0199254. doi: 10.1371/journal.pone.0199254 (PMC6021101; doi:10.1371/journal.pone.0199254)
Supplement: S2 Table — (DOCX) [file pone.0199254.s009.docx]

**Supporting information**

Table S2. Tissue Microarray Data.

| Sample | Subtype | Positivity |
| --- | --- | --- |
| 324 | Luminal A | 0.200955 |
| 327 | Luminal A | 0.138381 |
| 330 | Luminal A | 0.0854259 |
| 333 | Luminal A | 0.0716903 |
| 338 | Luminal A | 0.097223 |
| 343 | Luminal A | 0.19001 |
| 345 | Luminal A | 0.142964 |
| 349 | Luminal A | 0.139304 |
| 350 | No Characterize | 0.047292 |
| 352 | Luminal A | 0.141894 |
| 353 | Luminal A | 0.032914 |
| 356 | No Characterize | 0.0246497 |
| 358 | Basal like | 0.084544 |
| 363 | Luminal A | 0.088143 |
| 365 | Luminal A | 0.139037 |
| 368 | Luminal A | 0.0338055 |
| 373 | Luminal A | 0.000353607 |
| 376 | No Characterize | 0.0264951 |
| 378 | Luminal A | 0.00994537 |
| 390 | Basal like | 0.0313474 |
| 391 | Luminal B | 0.0368374 |
| 395 | HER2+ | 0.0311991 |
| 398 | HER2+ | 0.168314 |
| 399 | HER2+ | 0.0328737 |
| 404 | Luminal A | 0.0245119 |
| 409 | Luminal B | 0.0267167 |
| 412 | Basal like | 0.0244121 |
| 413 | Luminal A | 0.0563073 |
| 419 | Basal like | 0.00127723 |
| 422 | Basal like | 0.00332072 |
| 423 | Basal like | 0.0309486 |
| 426 | Basal like | 0.111533 |
| 428 | Luminal A | 0.0416148 |
| 429 | Luminal A | 0.0855761 |
| 431 | Luminal A | 0.0554005 |
| 432 | No Characterize | 0.0602915 |
| 435 | Basal like | 0.0339867 |
| 436 | Luminal A | 0.085914 |
| 437 | Luminal A | 0.0387797 |
| 438 | HER2+ | 0.0662766 |
| 440 | Luminal A | 0.0707441 |
| 441 | Luminal A | 0.0655495 |
| 451 | No Characterize | 0.07007 |
| 452 | Luminal A | 0.015058 |
| 455 | Basal like | 0.0154746 |
| 457 | HER2+ | 0.0104355 |
| 465 | No Characterize | 0.0440847 |
| 467 | Luminal A | 0.0315715 |
| 469 | No Characterize | 0.0129676 |
| 471 | Luminal A | 0.0148399 |
| 472 | No Characterize | 0.0531222 |
| 475 | Basal like | 0.0314746 |
| 476 | No Characterize | 0.00902974 |
| 479 | Luminal A | 0.164058 |
| 480 | Luminal A | 0.00925724 |
| 486 | No Characterize | 0.0300404 |
| 487 | Luminal A | 0.0694108 |
| 490 | Luminal A | 0.0270258 |
| 491 | Luminal B | 0.140788 |
| 493 | No Characterize | 0.026304 |
| 494 | HER2+ | 0.0449184 |
| 506 | Luminal A | 0.0265569 |
| 509 | Basal like | 0.00712733 |
| 511 | No Characterize | 0.0370104 |
| 515 | Luminal A | 0.0330487 |
| 519 | Luminal A | 0.112267 |
| 521 | Luminal A | 0.0348132 |
| 523 | Luminal A | 0.0218287 |
| 524 | No Characterize | 0.0799659 |
| 525 | Luminal A | 0.054666 |
| 527 | Luminal A | 0.0106196 |
| 528 | Luminal B | 0.0266403 |
| 534 | Luminal A | 0.0183615 |
| 535 | No Characterize | 0.017715 |
| 541 | HER2+ | 0.0985477 |
| 545 | Luminal A | 0.0185883 |
| 547 | Luminal A | 0.0653422 |
| 548 | Luminal A | 0.0239645 |
| 557 | Luminal A | 0.0907857 |
| 567 | No Characterize | 0.053668 |
| 569 | No Characterize | 0.0378118 |
| 570 | HER2+ | 0.0517933 |
| 573 | No Characterize | 0.0389383 |
| 575 | Luminal A | 0.0396854 |
| 577 | Luminal B | 0.0363607 |
| 579 | Basal like | 0.0227654 |
| 580 | Basal like | 0.0452366 |
| 585 | Luminal A | 0.0432819 |
| 590 | Basal like | 0.0293457 |
| 594 | Luminal A | 0.0199464 |
| 598 | Luminal A | 0.149649 |
| 600 | Basal like | 0.0111264 |
| 606 | Basal like | 0.0505398 |
| 610 | Basal like | 0.0113942 |
| 616 | Luminal A | 0.0940654 |
| 619 | Basal like | 0.012508 |
| 620 | HER2+ | 0.0329266 |
| 629 | Luminal A | 0.0222971 |
| 631 | Luminal A | 0.0191177 |
| 641 | Basal like | 0.0952506 |
| 643 | Luminal A | 0.0220656 |
| 644 | Luminal A | 0.0552013 |
| 646 | Luminal A | 0.0167699 |
| 647 | Luminal A | 0.138632 |
| 650 | Luminal A | 0.088069 |
| 664 | Luminal A | 0.295443 |
| 667 | Luminal A | 0.0215156 |
| 668 | Luminal A | 0.0108193 |
| 669 | Luminal A | 0.0211998 |
| 671 | No Characterize | 0.0895316 |
| 673 | Basal like | 0.0313065 |
| 674 | Basal like | 0.187679 |
| 676 | Luminal A | 0.0853566 |
| 678 | Luminal A | 0.0125382 |
| 680 | No Characterize | 0.0359741 |
| 683 | Luminal A | 0.0231216 |
| 691 | Luminal A | 0.0625572 |
| 697 | No Characterize | 0.0845387 |
| 707 | No Characterize | 0.0278165 |
| 713 | Basal like | 0.0445373 |
| 729 | Luminal A | 0.0285326 |
| 737 | Luminal A | 0.173598 |
| 741 | Luminal A | 0.0236935 |
| 749 | Luminal A | 0.0213338 |
| 751 | No Characterize | 0.0283657 |
| 757 | No Characterize | 0.00706878 |
| 759 | No Characterize | 0.0254232 |
| 761 | Luminal B | 0.0653114 |
| 763 | Basal like | 0.0185502 |
| 768 | Basal like | 0.051881 |
| 771 | Luminal A | 0.0479577 |
| 772 | Luminal A | 0.02606 |
| 778 | Luminal A | 0.0286033 |
| 779 | Luminal A | 0.0224534 |
| 781 | Luminal A | 0.0481192 |
| 787 | Luminal A | 0.318991 |
| 789 | Luminal A | 0.0378299 |
| 791 | Luminal A | 0.0161437 |
| 793 | No Characterize | 0.0422721 |
| 795 | Luminal A | 0.0182709 |
| 798 | Luminal A | 0.173483 |
| 802 | Luminal A | 0.0649488 |
| 805 | Luminal A | 0.0173982 |
| 809 | Luminal A | 0.0478133 |
| 817 | No Characterize | 0.0464156 |
| 818 | Luminal A | 0.0476152 |
| 819 | Basal like | 0.0477369 |
| 832 | Luminal A | 0.00987928 |
| 834 | Basal like | 0.0931598 |
| 835 | No Characterize | 0.041778 |
| 853 | No Characterize | 0.0642798 |
| 867 | Luminal A | 0.0116781 |
| 871 | Luminal A | 0.0351062 |
| 873 | Luminal A | 0.0302157 |
| 875 | HER2+ | 0.0566138 |
| 877 | Luminal A | 0.0197834 |
| 878 | Luminal A | 0.0361914 |
| 885 | Basal like | 0.055646 |
| 887 | Luminal A | 0.0451845 |
| 888 | Luminal A | 0.0594895 |
| 958 | Basal like | 0.0324595 |
| 959 | Luminal A | 0.0191224 |
| 962 | Luminal A | 0.00694943 |
| 965 | HER2+ | 0.050172 |
| 968 | Luminal B | 0.0425261 |
| 979 | Luminal A | 0.00734358 |
| 992 | Luminal B | 0.163577 |
| 994 | No Characterize | 0.0383381 |
| 995 | No Characterize | 0.00629534 |
| 996 | Luminal A | 0.0361499 |
| 998 | Luminal A | 0.0239358 |
| 1002 | Luminal A | 0.0165363 |
| 1005 | No Characterize | 0.0645851 |
| 1012 | HER2+ | 0.0038969 |
| 1016 | Luminal A | 0.00903063 |
| 1018 | Luminal A | 0.100895 |
| 1019 | Luminal A | 0.0153609 |
| 1021 | Luminal A | 0.0519691 |
| 1029 | Luminal A | 0.0101839 |
| 1032 | No Characterize | 0.0048731 |
| 1033 | Luminal A | 0.0125659 |
| 1034 | Luminal A | 0.0285125 |
| 1036 | Luminal A | 0.0522294 |
| 1038 | Luminal B | 0.0390774 |
| 1039 | HER2+ | 0.0722957 |
| 1042 | Basal like | 0.0138157 |
| 1044 | No Characterize | 0.00794584 |
| 1049 | Luminal A | 0.0486088 |
| 1053 | No Characterize | 0.0177673 |
| 1061 | Luminal A | 0.0303081 |
| 1062 | HER2+ | 0.0187697 |
| 1068 | Luminal A | 0.0281968 |
| 1075 | Luminal B | 0.0131899 |
| 1076 | Luminal A | 0.0519884 |
| 1077 | Luminal A | 0.00763057 |
| 1078 | No Characterize | 0.0796982 |
| 1081 | Luminal A | 0.0175328 |
| 1086 | Luminal A | 0.0603958 |
| 1095 | Luminal A | 0.0114604 |
| 1099 | Luminal A | 0.027526 |
| 1101 | Luminal A | 0.0269647 |
| 1102 | Luminal A | 0.0207583 |
| 1103 | Luminal A | 0.0089215 |
| 1110 | Luminal A | 0.0164173 |
| 1111 | Luminal A | 0.0268536 |
| 1112 | No Characterize | 0.00977581 |
| 1113 | Luminal A | 0.00286894 |
| 1114 | Luminal A | 0.0188983 |
| 1115 | Luminal A | 0.121816 |
| 1117 | Luminal A | 0.0254182 |
| 1118 | No Characterize | 0.0029107 |
| 1119 | Luminal A | 0.0242916 |
| 1124 | Luminal A | 0.00806433 |
| 1125 | No Characterize | 0.00806231 |
| 1135 | Luminal A | 0.0376088 |
| 1136 | Luminal A | 0.00725052 |
| 1139 | Luminal A | 0.0305384 |
| 1140 | Luminal A | 0.0542212 |
| 1141 | No Characterize | 0.00614277 |
| 1143 | Basal like | 0.0226912 |
| 1145 | Luminal A | 0.0282531 |
| 1147 | Luminal A | 0.0301669 |
| 1150 | No Characterize | 0.101315 |
| 1153 | Basal like | 0.0508936 |
| 1155 | Basal like | 0.0102486 |
| 1156 | Basal like | 0.0169433 |
| 1157 | Luminal A | 0.0441407 |
| 1159 | Luminal A | 0.0350502 |
| 1160 | Luminal A | 0.026003 |
| 1161 | Luminal A | 0.014041 |
| 1162 | Luminal A | 0.00893349 |
| 1164 | Luminal A | 0.0130015 |
| 1165 | Luminal A | 0.0264107 |
| 1166 | No Characterize | 0.0339505 |
| 1168 | Luminal A | 0.019333 |
| 1169 | No Characterize | 0.0324312 |
| 1171 | HER2+ | 0.00751395 |
| 1172 | HER2+ | 0.0102349 |
| 1176 | No Characterize | 0.0161051 |
| 1179 | No Characterize | 0.387512 |
| 1180 | Luminal B | 0.0093375 |
| 1181 | HER2+ | 0.0590927 |
| 1187 | Luminal A | 0.02903 |
| 1189 | Luminal A | 0.00992118 |
| 1190 | Luminal A | 0.0354358 |
| 1191 | Basal like | 0.0197428 |
| 1193 | Luminal A | 0.0185965 |
| 1194 | Luminal A | 0.105161 |
| 1195 | HER2+ | 0.0328419 |
| 1199 | Luminal A | 0.0292517 |
| 1200 | Luminal A | 0.0157631 |
| 1203 | Luminal A | 0.0151874 |
| 1204 | Basal like | 0.0262557 |
| 1206 | Luminal A | 0.047629 |
| 1207 | No Characterize | 0.0149148 |
| 1208 | Basal like | 0.0165509 |
| 1209 | Luminal B | 0.0552199 |
| 1210 | No Characterize | 0.0109655 |
| 1214 | Luminal A | 0.0311418 |
| 1217 | Luminal A | 0.0148043 |
| 1218 | Luminal A | 0.0223454 |
| 1219 | HER2+ | 0.0275218 |
| 1220 | Basal like | 0.137906 |
| 1222 | Luminal A | 0.0161915 |
| 1229 | Luminal A | 0.0103448 |
| 1233 | Luminal A | 0.076789 |
| 1236 | Luminal A | 0.00888901 |
| 1239 | Luminal A | 0.367351 |
| 1240 | Luminal A | 0.0156549 |
| 1246 | Luminal B | 0.0404749 |
| 1255 | HER2+ | 0.0585198 |
| 1260 | HER2+ | 0.03325 |
| 1262 | Luminal A | 0.0143268 |
| 1264 | HER2+ | 0.0212353 |
| 1267 | Luminal A | 0.0216115 |
| 1271 | Basal like | 0.0185396 |
| 1274 | Luminal A | 0.131463 |
| 1275 | Luminal A | 0.0344618 |
