# Supplementary material for: High CD90 (THY-1) expression positively correlates with cell transformation and worse prognosis in basal-like breast cancer tumors
Source: PLoS One. 2018 Jun 27;13(6):e0199254. doi: 10.1371/journal.pone.0199254 (PMC6021101; doi:10.1371/journal.pone.0199254)
Supplement: S3 Table — (DOCX) [file pone.0199254.s010.docx]

**Supporting information**

Table S3. Correlation of CD90 expression with clinicopathological and molecular features of human invasive ductal carcinomas.

| **Patient characteristics** | | **CD90 High** | **CD90 Low** | **P-value** |
| --- | --- | --- | --- | --- |
|  |  |  |  |  |
| Age at diagnosis | <50 | 38 | 61 | 0.119 |
|  | >= 50 | 51 | 128 |  |
|  |  |  |  |  |
| Tumor size | <3.5 | 39 | 85 | 0.959 |
|  | >=3.5 | 50 | 104 |  |
|  |  |  |  |  |
| Perou/Solie classification |  |  |  |  |
|  | Luminal | 57 | 115 |  |
|  | HER2 | 9 | 13 | 0.638 |
|  | Basal like | 11 | 27 |  |
|  |  |  |  |  |
| Lymph node status | N0 | 31 | 64 |  |
|  | N1 | 27 | 62 | 0.859 |
|  | N2 | 20 | 34 |  |
|  | N3 | 11 | 25 |  |
|  |  |  |  |  |
| Histological grade | 1 | 17 | 25 | 0.411 |
|  | 2 | 52 | 113 |  |
|  | 3 | 20 | 50 |  |
|  |  |  |  |  |
| Adjuvant therapy | None | 16 | 27 | 0.538 |
|  | Yes | 73 | 162 |  |
|  |  |  |  |  |
| ER status | Negative | 26 | 49 | 0.738 |
|  | Positive | 61 | 132 |  |
|  |  |  |  |  |
| PR status | Negative | 44 | 83 | 0.557 |
|  | Positive | 44 | 100 |  |
|  |  |  |  |  |
| HER2 | Negative | 65 | 136 | 0.717 |
|  | Positive | 13 | 22 |  |
|  |  |  |  |  |
