# Supplementary material for: High CD90 (THY-1) expression positively correlates with cell transformation and worse prognosis in basal-like breast cancer tumors
Source: PLoS One. 2018 Jun 27;13(6):e0199254. doi: 10.1371/journal.pone.0199254 (PMC6021101; doi:10.1371/journal.pone.0199254)
Supplement: S4 Table — (DOCX) [file pone.0199254.s011.docx]

**Supporting information**

Table S4. Cox TFU Correlation.

| Cox TFU |  |  |  |  |  |  |
| --- | --- | --- | --- | --- | --- | --- |
|  |  | **Variable** | **Hazard ratio** | **Lower 95%** | **Upper 95%** | **P-value** |
|  |  |  |  |  |  |  |
|  |  | Grade (ordinal) | 1.87 | 1.30 | 2.70 | 0.001 |
|  |  | Tumor size | 1.68 | 1.43 | 1.98 | <0.001 |
|  |  | Age at diagnosis | 1.08 | 0.89 | 1.32 | 0.435 |
|  | All data | ER status | 0.64 | 0.37 | 1.08 | 0.094 |
|  |  | PR status | 1.48 | 0.89 | 2.45 | 0.133 |
|  |  | Adjuvant therapy | 1.00 | 0.56 | 1.79 | 0.992 |
|  |  | CD90 | 1.18 | 0.98 | 1.42 | 0.079 |
|  |  |  |  |  |  |  |
|  |  |  |  |  |  |  |
|  |  |  |  |  |  |  |
|  |  | Grade (ordinal) | 2.14 | 1.32 | 3.48 | 0.002 |
|  |  | Tumor size | 1.91 | 1.47 | 2.48 | <0.001 |
|  | Luminal A | Age at diagnosis | 1.01 | 0.77 | 1.32 | 0.940 |
|  |  | Adjuvant therapy | 0.92 | 0.44 | 1.91 | 0.823 |
|  |  | CD90 | 1.05 | 0.83 | 1.33 | 0.676 |
|  |  |  |  |  |  |  |
|  |  |  |  |  |  |  |
|  |  |  |  |  |  |  |
|  |  | Grade (ordinal) | 0.76 | 0.30 | 1.95 | 0.574 |
|  |  | Tumor size | 1.37 | 0.95 | 1.97 | 0.095 |
|  | Basal-like | Age at diagnosis | 1.67 | 1.03 | 2.72 | 0.039 |
|  |  | Adjuvant therapy | 1.31 | 0.36 | 4.78 | 0.684 |
|  |  | CD90 | 1.78 | 1.05 | 3.01 | 0.032 |
|  |  |  |  |  |  |  |
|  |  |  |  |  |  |  |
