# Supplementary material for: High CD90 (THY-1) expression positively correlates with cell transformation and worse prognosis in basal-like breast cancer tumors
Source: PLoS One. 2018 Jun 27;13(6):e0199254. doi: 10.1371/journal.pone.0199254 (PMC6021101; doi:10.1371/journal.pone.0199254)
Supplement: S5 Table — (DOCX) [file pone.0199254.s012.docx]

**Supporting information**

Table S5. Cox MFS Correlation.

|  |  |  |  |  |  |  |
| --- | --- | --- | --- | --- | --- | --- |
|  |  | **Variable** | **Hazard ratio** | **Lower 95%** | **Upper 95%** | **P-value** |
|  |  |  |  |  |  |  |
|  |  | Grade (ordinal) | 1.95 | 1.35 | 2.81 | 0.000 |
|  |  | Tumor size | 1.80 | 1.51 | 2.14 | 0.000 |
|  |  | Age at diagnosis | 1.05 | 0.86 | 1.27 | 0.642 |
|  | All data | ER status | 0.69 | 0.42 | 1.15 | 0.154 |
|  |  | PR status | 1.39 | 0.85 | 2.26 | 0.188 |
|  |  | Adjuvant therapy | 1.01 | 0.57 | 1.79 | 0.977 |
|  |  | CD90 | 1.18 | 0.98 | 1.42 | 0.089 |
|  |  |  |  |  |  |  |
|  |  |  |  |  |  |  |
|  |  | Grade (ordinal) | 2.12 | 1.30 | 3.47 | 0.003 |
|  |  | Tumor size | 1.80 | 1.40 | 2.31 | <0.001 |
|  | Luminal A | Age at diagnosis | 0.97 | 0.74 | 1.27 | 0.822 |
|  |  | Adjuvant therapy | 0.97 | 0.47 | 2.02 | 0.936 |
|  |  | CD90 | 1.08 | 0.86 | 1.37 | 0.506 |
|  |  |  |  |  |  |  |
|  |  |  |  |  |  |  |
|  |  | Grade (ordinal) | 0.75 | 0.29 | 1.98 | 0.563 |
|  |  | Tumor size | 1.42 | 0.98 | 2.07 | 0.064 |
|  | Basal-like | Age at diagnosis | 1.58 | 1.00 | 2.51 | 0.052 |
|  |  | Adjuvant therapy | 1.28 | 0.35 | 4.63 | 0.711 |
|  |  | CD90 | 1.56 | 0.94 | 2.59 | 0.087 |
|  |  |  |  |  |  |  |
